# A genome-wide association meta-analysis implicates Hedgehog and Notch signaling in Dupuytren’s disease

**DOI:** 10.1038/s41467-023-44451-0

**Published:** 2024-01-03

**Authors:** Sophie A. Riesmeijer, Zoha Kamali, Michael Ng, Dmitriy Drichel, Bram Piersma, Kerstin Becker, Thomas B. Layton, Jagdeep Nanchahal, Michael Nothnagel, Ahmad Vaez, Hans Christian Hennies, Paul M. N. Werker, Dominic Furniss, Ilja M. Nolte

**Affiliations:** 1 University of Groningen, University Medical Center Groningen, Department of Plastic Surgery, Groningen, The Netherlands; 2 University of Groningen, University Medical Center Groningen, Department of Epidemiology, Groningen, The Netherlands; 3 Department of bioinformatics, School of Advanced Medical Technologies, Isfahan University of Medical Sciences, Isfahan, Iran; 4 Nuffield Department of Orthopaedics, Rheumatology, and Musculoskeletal Science, Botnar Research Centre, University of Oxford, Oxford, UK; 5 Cologne Center for Genomics, University of Cologne, Cologne, Germany; 6 Faculty of Medicine and the Cologne University Hospital, Cologne, Germany; 7 University of Groningen, Groningen, The Netherlands; 8 Kennedy Institute, University of Oxford, Oxford, UK; 9 Department of Biological Sciences, University of Huddersfield, Huddersfield, UK

**Keywords:** Genetic association study, Population genetics, Connective tissue diseases

## Abstract

Dupuytren’s disease (DD) is a highly heritable fibrotic disorder of the hand with incompletely understood etiology. A number of genetic loci, including Wnt signaling members, have been previously identified. Our overall aim was to identify novel genetic loci, to prioritize genes within the loci for functional studies, and to assess genetic correlation with associated disorders. We performed a meta-analysis of six DD genome-wide association studies from three European countries and extensive bioinformatic follow-up analyses. Leveraging 11,320 cases and 47,023 controls, we identified 85 genome-wide significant single nucleotide polymorphisms in 56 loci, of which 11 were novel, explaining 13.3–38.1% of disease variance. Gene prioritization implicated the Hedgehog and Notch signaling pathways. We also identified a significant genetic correlation with frozen shoulder. The pathways identified highlight the potential for new therapeutic targets and provide a basis for additional mechanistic studies for a common disorder that can severely impact hand function.

## Abstract (web summary)

In this meta-analysis of the fibrotic hand disorder Dupuytren’s disease, the authors identify genetic risk variants, study them for their functional consequences and implicate two signaling pathways involved in the disease mechanism of this debilitating disorder.

## Introduction

Fibrosis, the excessive accumulation of extracellular matrix components, can affect nearly every tissue in the body and is increasingly recognized as a major cause of morbidity and mortality^1^. Dupuytren’s disease (DD) is a fibrotic disorder of the fascias of the hand that causes fingers to irreversibly contract. It is also associated with excess mortality due to a wide range of causes, including cancer^2^. DD is very common and its prevalence increases with age, affecting 12% of Western population aged 55 years to 29% of those aged 75 years^3^. The initial presentation is as a highly cellular nodule that progresses to form fibrous cords. As the cords extend into the finger and undergo remodeling and shortening, patients develop contractures that impair function and quality of life^4^. Treatment of DD is currently limited to late-stage disease to reduce flexion contractures by disrupting or excising the cords. However, recurrence rates are high, and there is no cure for this debilitating disease^5^.

Current understanding of the etiology of DD is limited, with a complex interaction between genetic and environmental factors^6^. There is strong evidence for an association with advanced age, male sex, family history of DD, heavy alcohol consumption, cigarette smoking, and manual work exposure^7^. Furthermore DD shares a genetic etiology with body mass index (BMI), type II diabetes mellitus (T2D), and levels of triglycerides and high-density lipoprotein (HDL)^8^. Intriguingly, adiposity is causally protective against DD^9,10^. Genome-wide associations studies (GWASs) have so far identified 61 genetic risk variants^11–13^. The overall (broad-sense) heritability of DD was estimated to be 80%, of which 67% is attributable to common genetic variants^8,14^. However, the variance explained by known genetic variants was estimated as 11.3%^12^. Thus, much of the genetic susceptibility for DD remains to be elucidated.

Development of novel therapeutic approaches requires a systematic approach of identification of novel genetic risk factors, broadening our understanding of the mechanisms involved with these genetic susceptibility loci, and studying their functional consequences. Here we addressed these aims by performing meta-analysis of GWAS data from six cohorts to prioritize genes involved in DD.

## Results

### Study cohorts

Sample and genotyping details of the cohorts included in the meta-GWAS are provided in Supplementary Data 1. An overview of the QC output parameters of the GWAS per cohort is given in Supplementary Data 2.

### Association analysis

**Fig. 1:**
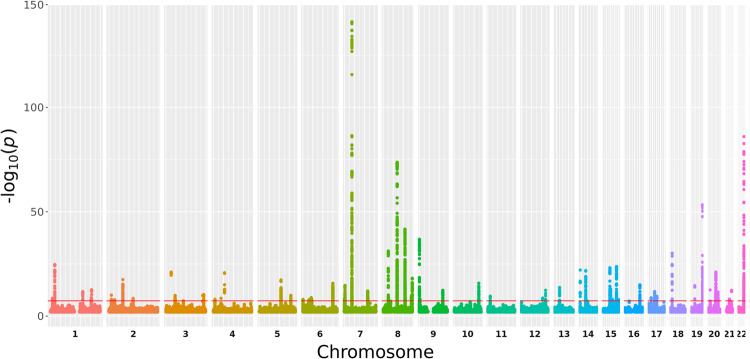
Manhattan plot of the logistic regression for the meta-GWAS association analysis. The horizontal red line represents a *p*-value threshold of 5 × 10^−8^ (e.g. the multiple comparison correction for genome-wide significance).

**Fig. 2:**
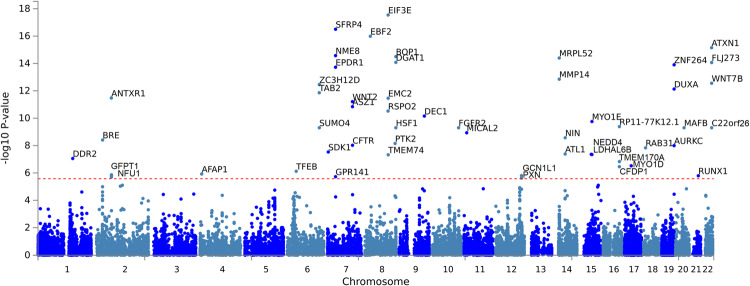
Gene-based analysis as computed by MAGMA, using a multiple regression model. Meta-GWAS summary statistics were mapped to 18,879 protein coding genes. Genome-wide significance (red dashed line in the plot) was defined by Bonferroni correction at *p* = 0.05/18,879 = 2.65 × 10^−6^.

The meta-analysis included a total of 11,320 cases and 47,023 controls, tested at 8,123,121 variants after QC. Clumping analysis identified 85 independent genome-wide significantly associated SNPs in 56 loci (Fig. 1; Supplementary Data 3; Supplementary Fig. 2). Forty-five of these loci have been previously reported^12,15^, 11 represent new loci. In addition, we identified 24 new secondary hits in 12 known loci. All 26 previously identified loci from the previous (UK) GWAS^12^ were confirmed, but for six loci the effects were found to be heterogeneous between the six cohorts and were therefore excluded from our meta-GWAS (Supplementary Data 4). We also found associations with 45 of the 61 loci implicated by Agren et al.^13^. Forest plots of the significantly associated SNPs are shown in Supplementary Fig. 3. Thirty-four of the 85 genome-wide significant meta-GWAS SNPs were replicated in the FinnGen cohort and 77 reached genome-wide significance in the combined meta-analysis with FinnGen (Supplementary Data 5). Five of the 85 genome-wide significant SNPs were not available in FinnGen. Gene-based analysis is shown in Fig. 2.

### Bioinformatic follow-up analyses

#### In silico annotation

In silico sequencing analysis returned 4542 and 2111 variants in moderate (*r*^2^ > 0.5) and high (*r*^2^ > 0.8) LD, respectively, with the identified 85 meta-GWAS SNPs (Supplementary Data 6). One of the meta-GWAS SNPs (rs1042704) as well as eight correlated SNPs were non-synonymous. Out of these eight nsSNPs, three were in high LD (*r*^2^ > 0.8) with two meta-GWAS SNPs: rs366905 and rs34412930. The nine nsSNPs mapped to the genes *TMEM81*, *DSTYK*, *SUMO4*, *CFTR*, *TNC*, *MMP14*, and *LDHAL6B* (three nsSNPs). We flagged four nsSNPs in *TMEM81, DSTYK, MMP14*, and *LDHAL6B* as deleterious based on scaled CADD scores >20. This score means that these missense mutations are among the top 1% of all possible substitutions in the human reference genome (~8.6 billion) ranked by deleteriousness based on over 60 different annotation sources^16^. This score is also larger than the median for all non-synonymous variations (i.e. 15).

In silico pleiotropy analysis (lookups of associated phenotypes) of all meta-GWAS SNPs and their correlated SNPs revealed associations with hematologic and certain anthropometric traits (Supplementary Data 6). Interestingly, two SNPs were associated with keloid, another fibroproliferative disorder influenced by transforming growth factor beta (TGF-β1) production and Wingless and Int-1 (Wnt) signaling^17^. Furthermore, rs11581010 has been previously associated with lower BMI^18^. Additional associations were found with variance of red cell counts (red cell distribution width), heel bone mineral density, and type II diabetes mellitus. The locus harboring *ZBTB40* was the most pleiotropic region, with reported GWAS associations with a variety of hematologic traits, bone mineral density traits, and chronic inflammatory diseases.

#### Gene prioritization, pathway, and tissue prioritization analyses

The full gene prioritization analyses can be found in the Supplementary Results. Since effect sizes of blood and fibroblast eQTLs showed a high similarity (*r* = 0.71; Supplementary Fig. 4), blood eQTL data were used, given the much larger available sample size. A list of 119 prioritized genes resulted from these analyses (Supplementary Data 7). These genes were then assessed for association of differential gene expression in fibroblasts and further functional analyses. The FINEMAP analysis showed that most of the 56 loci the index SNPs had considerable evidence for being causal themselves and that the proportion of variants with a considerable evidence was the highest for exonic variants (see Supplementary Data 8 and Supplementary Fig. 10).

Functional enrichment analysis showed that our results were enriched for genes involved in abnormal limb, cartilage, and skeleton morphology (Supplementary Data 9). Further functional analysis suggested deeper mechanistic insights, most importantly TGF-β signaling, epithelial cell migration and cell-matrix adhesion (Supplementary Data 10). Protein-protein interaction network of the prioritized genes revealed two connected components, of which the first one was suggestive of response to stress and the second one was suggestive of viral/bacterial infection (Supplementary Data 24).

Tissue prioritization results showed enrichment of 12 tissues for the expression of DD prioritized genes (FDR < 0.01; Supplementary Data 11), on top of which fibroblasts showed the highest expression levels (Supplementary Fig. 5).

#### Transcriptome-wide association study

SMR analysis with fibroblast eQTLs revealed seven genes with expression levels significantly associated with DD (*P*_SMR_ < 6.84 × 10^−6^)^19^. Four of these associations remained significant after the heterogeneity test (*P*_HEIDI_ ≥ 7.14 × 10^−3^, Supplementary Data 12). These include *RPLP0*, with a higher risk for DD, and *CTD-2587M2.1*, *DLG5*, and *TEAD3* with predicted protective effects against DD. The long non-coding RNA *CTD-2587M2.1* was associated in SMR analysis with blood eQTLs, with consistent direction of effect (Supplementary Data 13).

Of the 119 prioritized genes for DD, 14 reached Bonferroni-corrected significance in the fibroblast SMR analysis (Supplementary Data 7). Five of these (*TNC, AFAP1, CHSY1, NEDD4*, and *CFDP1*) were identified in at least three bioinformatic follow-up analyses and fibroblast SMR analysis.

#### Cell population-relevant genes

**Fig. 3:**
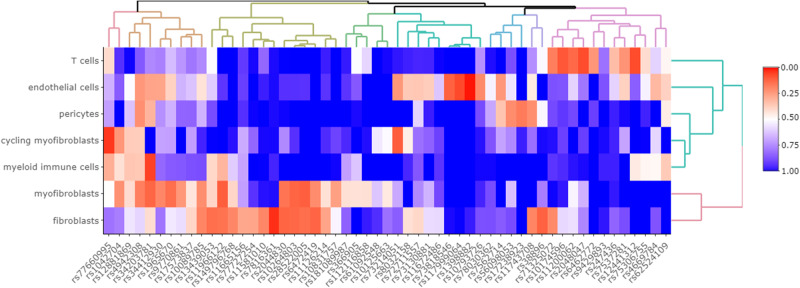
Cell population-relevant genes. Heatmap showing specificity scores (between 0 [red] and 1 [blue] where a lower value indicates greater specificity to the cell type), produced by SNPsea, of meta-GWAS associated DD loci in cell populations derived from single cell RNA-seq of DD nodules (*n* = 6 patients)^60^. Dendrograms (clustering trees) can be observed for cell populations (y-axis) as well as genetic loci (x-axis).

Next, we aimed to identify cell types whose function is likely to be influenced by genes in our risk loci. Of the 85 meta-GWAS hits, SNPsea was unable to identify SNPs rs12442366 and rs886423. Twelve loci did not contain any genes. SNPsea merges SNPs with shared genes into single loci to avoid multiple counting of genes, thus resulting in a dataset of 55 gene sets. Myofibroblasts showed the strongest association to the meta-GWAS risk loci (*p* = 0.08). Moreover, automatic clustering highlighted that myofibroblasts and fibroblasts have more similar expression of genes in our meta-GWAS loci than other cell types (Fig. 3). In Supplementary Data 14 genes with the greatest specificity to each cell population are given for each combination of SNP and cell type.

### Polygenic risk scores

**Fig. 4:**
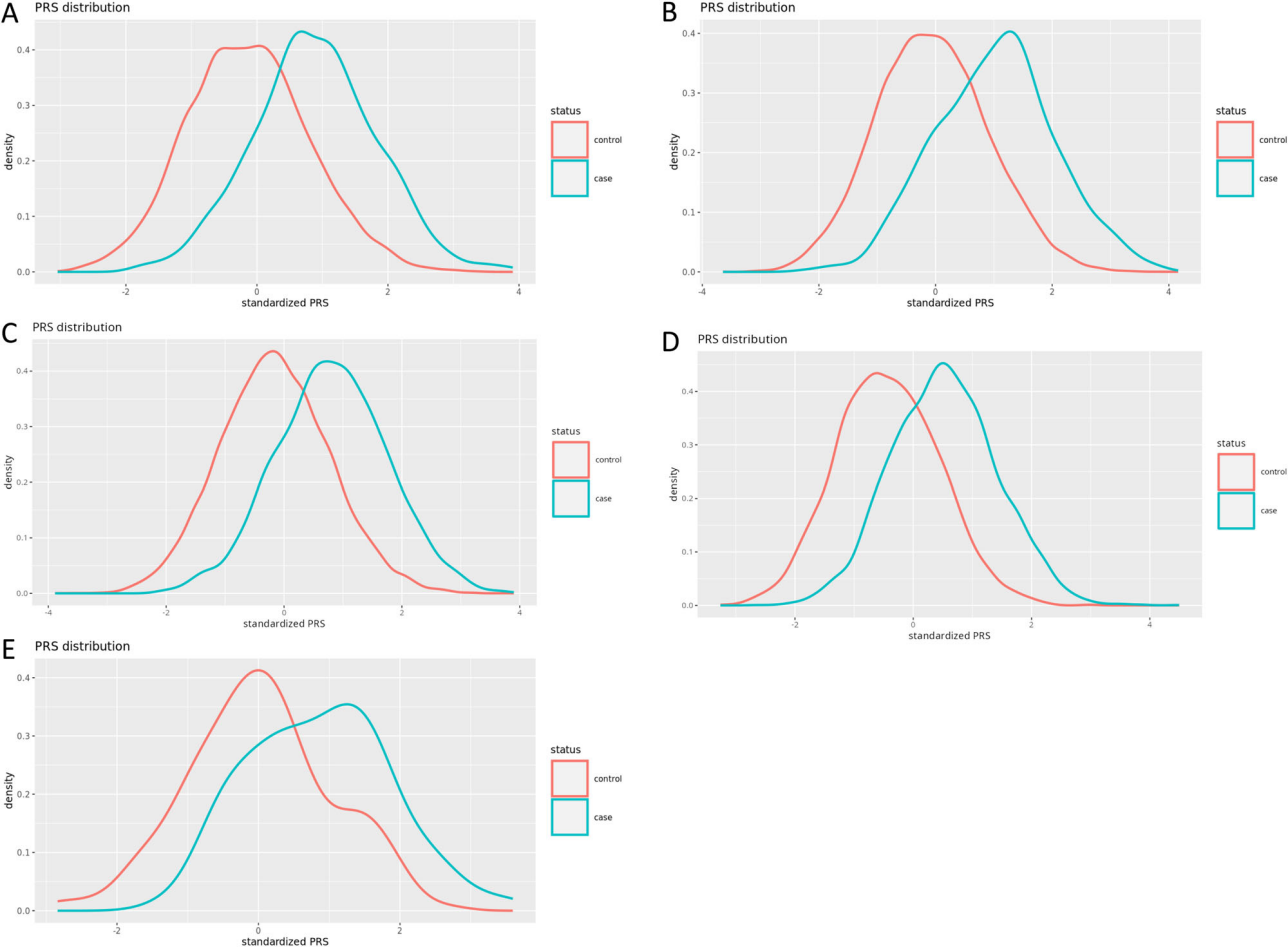
Variance explained through polygenic risk score analyses. Density plot of the PRS distribution for cases and controls in (**A**) the Dutch CytoSNP cohort, (**B**) the Dutch GSA cohort, (**C**) UK Biobank cohort (**D**) the UK BSSH-GODD cohort, and (**E**) the Dutch FinnGen cohort.

The distributions of standardized PRS were in highly significantly different between cases and controls for both Dutch cohorts (CytoSNP OR = 3.25, 95% CI = 2.92-3.62, *p* = 2.0 × 10^−102^; GSA OR = 3.16, 95% CI = 2.87-3.48, *p* = 3.9 × 10^−122^; Fig. 4) and for both UK cohorts (UK Biobank OR = 2.94, 95% CI = 2.82-3.07, *p* = 1.0 × 10^−310^; BSSH-GODD OR = 3.48, 95% CI = 3.26-3.72, *p* = 1.9 × 10^−307^; Fig. 4) Liability-adjusted Nagelkerke’s pseudo *R*^2^ measures showed that 13.3% of the variance can be explained by the PRS in the Dutch CytoSNP cohort, 15.3% in the Dutch GSA cohort, 30.0% in the UK Biobank cohort, and 38.1% in the BSSH-GODD cohort. Very recently the Dutch Lifelines cohort was further genotyped using another array (FinnGen Thermo Fisher Axiom® custom array) for 28,500 additional participants, of whom 110 reported to have DD by questionnaire and 6,148 unrelated individuals were selected as controls. For this cohort, the PRS was less significant (OR = 2.44, 95% CI = 1.74-3.43, *p* = 2.3 × 10^−7^) and the liability-adjusted Nagelkerke’s pseudo *R*^2^ was 13.3%.

### Genetic correlations

Frozen shoulder showed a significant positive genetic correlation of 0.30 with DD (*p*-value = 1.9 × 10^−6^), suggesting that there is an overlap between causal variants for DD and for frozen shoulder (Supplementary Data 15). Increasing BMI was significantly negatively correlated to DD (*p*-value = 1.2 × 10^−8^, *r* = −0.14), and increasing HDL was positively correlated (*p*-value = 3.6 × 10^−5^, *r* = 0.12). Genetic correlations for fasting glucose, HbA1c, triglycerides, idiopathic pulmonary fibrosis, psoriasis, systemic sclerosis, T2D, bone mineral density, and height with DD were not significant.

### Colocalization analysis

The significantly genetically correlated traits frozen shoulder, BMI, and HDL were considered for colocalization analysis. For frozen shoulder, five genome-wide significant SNPs have been identified^20^. Colocalization analysis revealed that DD and frozen shoulder share the same causal variants rs1042704 (posterior probability 0.99) and rs28606049 (posterior probability 0.99). SNP rs2472660, which was also located in a locus associated to DD, was not found to be causally related to both disorders. For BMI, two previously associated loci were within a locus also associated with DD: rs10779751 and rs7607490^21^. For rs10779751, neighboring SNP rs11581010 was revealed as a shared causal variant for both DD and BMI (posterior probability 0.99). For rs7607490, none of the SNPs within its region had a causal effect on both BMI and DD (posterior probability 0.12). None of the previously associated HDL SNPs were at the same loci as genome-wide significant DD SNPs, thus colocalization analysis for HDL could not be performed.

### Other gene prioritization analyses

Results from supplementary methods and results are available in the supplements: list of prioritized genes, Supplementary Data 17; co-regulation analysis, Supplementary Data 18; multi-layer analysis, Supplementary Data 19; multi-QTL analysis, Supplementary Data 20; functional enrichment analysis of 73 prioritized genes, Supplementary Data 21; functional enrichment analysis of 23 prioritized genes, Supplementary Data 22; functional enrichment analysis of 119 prioritized genes, Supplementary Data 23 Protein-protein interaction (composite) network analysis, Supplementary Data 24.

## Discussion

Although DD is a common disease with a strong genetic component, underlying genetic factors and disease mechanisms are widely elusive. Therefore, the aims of this study were to identify additional genetic loci and pathways for DD to further explain the genetic variance of DD, and to provide directions for future functional follow-up studies. We performed the largest GWAS of DD to date, meta-analyzing six cohorts, including 11,320 cases and 47,023 controls. It yielded 85 genome-wide significantly associated SNPs at 56 loci, 34 of which have not been previously described. With these variants thrice the amount of phenotypic variance (narrow-sense heritability) can be explained, up to 38.1% in contrast to 11.3%^12^. Nevertheless, a large amount remains undiscovered considering the estimated heritability of 80%^8,12,14^.

Replication of our genome-wide significant SNPs in the FinnGen cohort revealed only a moderate number of replicated SNPs (34 out of 85). This moderate overlap of SNPs available from both datasets might be explained by the fact that the Finnish population is known to have experienced a population bottleneck and therefore to be genetically diverse from other European populations^22^. Consequently we chose to report all 85 SNPs as DD associated variants and not only those that could be replicated in FinnGen. In addition, FinnGen used a ThermoFisher Axiom custom array containing variants enriched in Finland, thereby explaining why many of the suggestive associations found by FinnGen were not available in our meta-GWAS. Nevertheless, the majority (*n* = 77) of the 85 genome-wide significant SNPs from our meta-GWAS reached a genome-wide significance in the meta-analysis of our meta-GWAS results and those from the FinnGen cohort.

Very recently, a meta-analysis of DD GWAS was performed using the results from three biobanks (UK Biobank, FinnGen, and Michigan Genomics Initiative)^13^. For this study, the authors identified DD cases based on available International Classification of Diseases codes (ICD, Ninth Revision, Clinical Modification; code 728.71) available from hospital inpatient data. In the current study, we used DD cases diagnosed and/or treated by a plastic or hand surgeon in our outpatient clinics. Due to the difference in diagnosing, these cohorts are likely not quite comparable regarding phenotyping, in addition to the abovementioned genetic diversity of the studied populations. We thus chose not to include these cohorts in our meta-analysis, but rather compare the results. From the 56 regions that we identified, Agren et al. also implicated 45, among which 25 that were already reported previously^11–13^. The eleven additional loci include the genes *MTOR, BABAM2, LRRC3B, LIMD1, LPP, AFAP1, LOC101927691, TFEB, MICAL2, MEOX1*, and *ADAMTS5*.

To prioritize genes that substantially impact DD phenotype we performed extensive bioinformatic follow-up analyses of our 85 genome-wide significantly associated SNPs in 56 loci in order to identify the most likely causal variants within these loci. These analyses identified 40 functional SNPs on the basis of it being a non-synonymous SNP, an eQTL, a multiQTL, or being colocalized with DD-related phenotypes (Supplementary Data 17. Although prioritized genes require experimental validation to reveal the mechanistic link with associated SNPs, they implicated the Hedgehog (Hh) and Notch signaling pathways. While Notch signaling has very recently been associated with DD^23^, we newly report on Hh signaling. Both may contain potential therapeutic drug targets. In addition, we also found further candidates in the Wnt/β-catenin and Hippo signaling pathways, which are already known to be involved in the pathogenesis of DD^11,12,24–26^. Therefore, these signaling pathways and their prioritized genes are discussed in more detail below and visualized schematically in Supplementary Fig. 6. We focus our discussion on genes identified in at least three gene prioritization analyses and replicated in the fibroblast SMR analysis (Supplementary Data 16), as we consider these results to be robust.

Hh signaling plays a crucial role in embryonic development, regulating differentiation, proliferation, and tissue patterning of the brain, internal organs, and limbs^27^. The importance of Hh signaling in fibrosis has already been established in both animal models and humans^27^. In humans, Hh signaling is implicated in fibrotic kidney disease, pancreatic fibrosis, liver fibrosis, and biliary fibrosis^28–30^. Hh signaling can be activated by injury^28–30^. Profibrotic factors such as TGF-β1 can activate expression of Hh members of the GLI family in human fibroblasts^31^. CHSY1 is a regulator of Hh signaling. It encodes an enzyme that plays a critical role in the biosynthesis of chondroitin sulfate, a glycosaminoglycan involved in many biological processes including cell proliferation and morphogenesis. To our knowledge we are the first to report a link between CHSY1 and fibrosis (see Supplementary Fig. 6). Future research on the role of *CHSY1* in DD should focus on studying its expression levels in DD myofibroblasts. The extracellular matrix (ECM) glycoprotein gene *TNC* (encoding tenascin C) is a target of the Hh signaling pathway transcription factors GLI1 and GLI2 (Supplementary Fig. 6)^27^. Berndt et al. found that tenascin C was one of the constituents of the extracellular matrix formed by Dupuytren’s myofibroblasts^32^. In kidney fibrosis tenascin C was induced by Sonic Hedgehog (SHH) and identified as a major constituent of promotion of fibroblast proliferation^33^. In our analyses tenascin C was found to be involved in one of the two major connected components of DD protein-protein interaction networks, which was enriched in ECM organization. As we also identified a nsSNP in *TNC*, functional studies might be particularly fruitful. DLG5 is a member of the MAGUK superfamily that is involved in maintenance of epithelial cell polarity, cell proliferation control, and cell migration and invasion^34^. In mice Dlg5 is required to interact with Hh receptor Smoothened (Smo) for Gli protein activation^35^. In mouse fibroblasts lacking Dlg5, Hh-induced Smo accumulation was observed^35^.

Several drugs targeting the Hh signaling pathway are being or have been developed, studied, and manufactured. Cyclopamine, a small molecule inhibitor of the transmembrane protein SMO, attenuated renal fibrosis in vivo^27^. Hh pathway inhibitors have been studied as cancer drugs^36–38^. Vismodegib also inhibits SMO, blocking activation of GLI proteins to transcribe Hh target genes. Thus, cyclopamine and vismodegib might have mitigating effects on the DD phenotype.

Notch signaling is a highly conserved embryonic developmental signaling pathway. It consists of several receptors, NOTCH1–NOTCH4, and their ligands, delta-like and jagged (JAG). Activation of the pathway usually occurs via expression of the ligand in a signal-giving cell^39^. Notch signaling has been shown to be activated in retinal, renal, and hepatic fibrosis^40–43^. In vessels of the microcirculation in DD nodules, pericytes (that surround the endothelial cells) were shown to specifically express NOTCH3^23^. Furthermore, the γ-secretase inhibitor XX (GSIXX, referred to as DBZ), a pharmacological inhibitor of Notch, effectively ameliorated renal fibrosis in mice^43^. *CHSY1* is a member of the Fringe family of genes that modulate Notch signaling via ligand interaction with Notch receptors^44^. Tian et al. described overproduction of JAG1 and subsequent Notch activation in absence of CHSY1 in fibroblasts from patients with syndromic brachydactyly associated with a truncating frameshift mutation in *CHSY1*^45^. Knockdown of CHSY1 promotes Notch signaling, and overexpression of CHSY1 reversed Notch activation^45^, consistent with the predicted protective effect on DD that we found for *CHSY1* in the SMR analysis with fibroblast data.

The Hippo signaling pathway has been previously implicated in fibrosis and DD^25,26^. We found a new association with *TEAD3*, a member of the TEA domain family of transcription factors that are essential in mediating YAP-dependent gene expression. YAP1 is a regulator of myofibroblast differentiation and contributes to the maintenance of the contractile phenotype in DD myofibroblasts^25^. As TEAD3 is a key transcription factor mediating YAP function^46^, we hypothesize that its decreased expression might up-regulate YAP dependent gene expression in DD.

We have demonstrated that the previously observed phenotypic association of frozen shoulder with DD^47^ likely results from a substantial genetic correlation. Moreover, we showed that frozen shoulder and DD share two causal variants among the three variants that are shared between the two diseases in total. Of these, rs1042704 is an exonic SNP in matrix metalloproteinase 14 gene (*MMP14)*, that has been shown to cause a specific defect in collagenolytic activity in DD derived fibroblasts^48^. Interestingly, in a series of 12 people treated for an inoperable gastric carcinoma with a synthetic matrix metalloproteinase inhibitor, half developed frozen shoulder or a condition resembling DD^49^. These findings further underline the importance of MMPs in both frozen shoulder and DD^48^. The second shared causal variant rs28606049 is an intron variant in *WNT7B*, which is highly upregulated in DD^24,50^. As inferred from the substantial genetic correlation, many more variants are likely shared between frozen shoulder and DD. We also reproduced previously described genetic correlations with BMI and HDL, but not for triglycerides and T2D. BMI and DD only shared one causal variant, indicating that LD between associated SNPs or (mediated) pleiotropic effects of non-genome-wide significant SNPs are likely the driving force behind the genetic correlation^51^. This is further underlined by our in silico pleiotropy analysis, where a substantial enrichment of DD loci in a previously conducted BMI GWAS was found. The same theory holds true for HDL and DD, as none of the genome-wide significant SNPs for HDL resided at the same loci as those for DD.

Strengths of this study include the large sample size of DD patients and controls and the thorough phenotyping of the DD patients by a plastic or hand surgeon, resulting in the identification of many additional loci. Furthermore, the inclusion of cohorts from multiple European countries achieved translatability of results across multiple European populations (except for Finns, as discussed above), instead of only one subpopulation. Moreover, genotype imputation facilitated integration of genotype data from multiple cohorts analyzed with different arrays and enhanced the power for detecting SNPs. We performed a multitude of bioinformatic follow-up analyses from different mechanistic angles and only took genes into account that were prioritized in at least three analyses and replicated in a tissue specific analysis. Therefore, we argue that the results are robust. Although we provided starting points for functional studies, a limitation of this study is that we did not investigate experimentally the effects of associated SNPs and prioritized genes in vitro. Unfortunately age and sex were not available for some cohorts. Therefore in these cohorts cases and controls could not be age and sex matched and these parameters could not be included in the analysis as covariates. However the result is likely that some statistical power is lost in case the controls were younger than the cases, as the control cohort may still include individuals who became cases after inclusion in the study. In case controls were older than cases, this could have introduced bias due to increased mortality, however this is likely not of great influence in the case of DD. In addition we excluded heterogeneous effects from the meta-analysis results, which will have filtered out a spurious result due to potential age or sex mismatching in one of the cohorts. Another limitation of this work is that we were not able to validate our meta-GWAS results in a genetically similar cohort, as the replication analysis used the FinnGen population which is genetically diverse from other European populations^22^. The generalizability of our results, acquired studying populations of European ancestry, to other ethnic populations, is likely limited. To our knowledge, no GWAS have been performed in populations other than from European ancestry. Increasing diversity of ancestries among GWAS study participants can advance our understanding of the genetic susceptibility to DD for all populations. We believe a next step in genetic epidemiological research into DD would be performing multi-ancestry GWAS, perhaps through utilizing multi-ethnic biobanking studies due to the lack of available cohorts.

In conclusion, this meta-analysis of six GWASs identified 34 novel loci for DD and newly implicated the Hh signaling pathway and confirmed association of the Notch signaling pathway in the etiology of DD. Prioritized genes *CHSY1, NEDD4*, and *DLG5* have regulatory properties in Notch, Hh, Hippo, and Wnt signaling. These pathways contain therapeutic targets for which a number of inhibitors exist. We have outlined starting points for future mechanistic studies for DD. Additionally, we found a genetic correlation between frozen shoulder and DD and identified two SNPs that are causal variants for both frozen shoulder and DD. Our data will help inform future mechanistic studies to validate therapeutic targets and develop new treatment strategies for DD. Until then, the increased knowledge about the genetic susceptibility to DD provided by this meta-GWAS facilitates research into individualized risk prediction for DD through genetic profiling.

## Methods

### Study cohorts

We used data from six cohorts with DD cases and healthy controls from the Netherlands, United Kingdom (UK), and Germany. The cases were individuals of European ancestry who had been diagnosed with DD by a plastic or hand surgeon and/or who had undergone surgical treatment for DD. Controls were population-based subjects from the Lifelines cohort study (the Netherlands), from the UK Household Longitudinal Study (UK), the UK Biobank initiative (UK), and from the PopGen and KORA studies (Germany) with no known diagnosis of DD. All study populations were described in detail previously^11,12,52–59^. All samples analyzed in previous GWASs^11,12,51,54,60^ were included in these cohorts.

### Ethical approval

The studies used in this meta-analysis were approved by the Research Ethics Committee or equivalent at all institutions where the data were collected: (1) The Genetic Origin of Dupuytren Disease (GODDAF) Study (the Netherlands) was approved by the Ethics Committee of the University Medical Center Groningen, document number 2007/067; (2) The Lifelines study (the Netherlands) was approved by the Ethics Committee of the University Medical Center Groningen, document number 2007/152. This study (‘The role of genetic variants in Dupuytren disease’) has Lifelines study ID OV18_0461; (3) The British Society for Surgery of the Hand Genetics of Dupuytren’s Disease (BSSH-GODD) study (United Kingdom) was approved by the Oxfordshire Research Ethics Committee, document number B/09/H0605/65; (4) The UK Biobank (United Kingdom) was approved by the North West Multi-Centre Research Ethics Committee, document number 11/NW/0382. This study (‘The Genetics and Epidemiology of Common Hand Conditions’) has UK Biobank study ID 22572; (5) The German Dupuytren Study was approved by the Ethics Commission of the Faculty of Medicine of the University of Cologne, document number 14/292. The KORA study was approved by the Ethics Committee of the Bavarian Medical Association (Bayerische Landesärztekammer) and the Bavarian commissioner for data protection and privacy (Bayerischer Datenschutzbeauftragter). The PopGen study was approved by the Ethical committee of the Medical Faculty of Christian-Albrechts-Universität (CAU), Kiel. Informed consent was obtained from all subjects in accordance with Declaration of Helsinki protocols.

### Genotyping, quality control and imputation procedures

The genotyping, quality control (QC), and imputation procedures of the UK and German cohorts were described in detail previously^12,51–53,60^ A detailed pipeline of genotype QC and imputation procedures for the Dutch case cohorts can be found in the Supplementary Materials.

### GWAS

GWAS for the Dutch cohorts were performed with logistic regression analysis in PLINK (version 1.9^61–64^. For each single nucleotide polymorphism (SNP) an analysis was performed with disease status as outcome and age, sex, and the first ten principal components as covariates. For the UK and German cohorts, for each SNP a logistic regression was performed with sex and principal components as covariates, calculated with PLINK (version 1.9) or SNPTEST (version 2.5.4-beta3), respectively^65,66^. Age was not available in these cohorts. The principal components were calculated using PLINK (version 1.9^61,64^. Quality control of the GWAS summary statistics was performed in R (version 3.6.1) using the GWASinspector package for each cohort separately^67^. In case of quality issues the respective cohort was notified and problems were solved. Using the QQ plots from GWASinspector, for each cohort specific imputation quality and allele frequency thresholds were set (see Supplementary Methods). Genomic reference build 37 (GRCh37/hg19) was used in this study.

### Meta-analysis

Meta-analysis of the six GWAS results (meta-GWAS) was performed using METAL with fixed effects inverse variance weighting method^68^. We used a double genomic control correction to control for genomic inflation due to population stratification within and between study cohorts^69^. After meta-analysis, only high-quality variants were considered for follow-up analyses: i.e. variants present in three or more (out of six) cohorts, variants present in >10,000 participants (~20% of total), and variants with a heterogeneity *p*-value > 0.05 (alongside other quality criteria) were considered for follow-up analyses. These filtered meta-GWAS summary statistics (containing only high-quality variants) were used for gene-based analysis through Functional Mapping and Annotation of Genome-Wide Association Studies (FUMA)^70^.

We sought to replicate significant loci discovered in our meta-GWAS in the FinnGen cohort consisting of 3248 cases and 197,724 controls and performed a combined meta-analysis for these loci of our meta-GWAS and FinnGen’s results^15^. For replication, SNPs with a one-sided *p*-value < 0.000588 (=0.05/85, i.e. Bonferroni correction) in FinnGen were considered replicated.

### Identifying independent loci and secondary signals

SNPs were regarded as genome-wide significant if the *p*-value was <5 × 10^−8^. Genome-wide significant loci were considered independent when SNPs in neighboring loci were separated by >1 Mb on the same chromosome or on different chromosomes. If a locus contained only one significant SNP, it was considered unreliable and was discarded. To ascertain secondary signals, clumping was performed in PLINK (*r*^2^ ≤ 0.01; distance < 1 Mb) at each genome-wide significantly associated genetic locus^65^.

### Bioinformatic follow-up approach

#### In silico annotation

To uncover functional characteristics of the independent genome-wide significant SNPs and their surrounding regions, we determined which SNPs were in at least moderate linkage disequilibrium (LD) (*r*^2^ > 0.5) with the GWAS SNPs based on 1000 G European reference data (with a maximum of 2 Mb distance) and annotated these using ANNOVAR (i.e. an in silico bioinformatics-based annotation approach)^71^. Variant call format (VCF) files for individuals of the European continental population from the 1000 Genomes Project, Phase 3 (version v5a, Feb. 20th 2015) were downloaded and data of the 2 Mb region surrounding each of the 85 identified SNPs were extracted with VCFtools^62,72^. The *r*^2^ between identified SNPs and all biallelic SNPs residing in the 2 Mb region was calculated with PLINK^65^. SNPs were deemed sufficiently correlated if *r*^2^ > 0.5. Identified and correlated SNPs were annotated using ANNOVAR (version October 2019) for functional consequences^73^. Nonsynonymous (ns, e.g. protein altering) SNPs were then characterized for their damaging effect using Combined Annotation Dependent Depletion (CADD) scores^16^. A scaled C-score > 20 was considered deleterious^16^. To better understand the possible function of the SNPs, we also performed an in silico pleiotropy analysis, that is, checking for association of all identified SNPs and SNPs in linkage disequilibrium (LD) (*r*^2^ > 0.5) with other traits and diseases available in the GWAS Catalog database (version 21 April 2021)^73,74^.

#### Gene prioritization, pathway, and tissue prioritization analyses

We followed up our GWAS results with a multi-omics post-GWAS approach^75,76^ to gain insight on the biology of DD phenotype and identify potential key players in disease pathogenesis (Supplementary Fig. 1, Supplementary Methods), including FINEMAP to identify the most likely causal variants within each locus^77^. We used blood eQTL data for two gene prioritization analyses (as a discovery) and fibroblast eQTL data as a replication analysis of significant discovery findings, as proposed by Qi et al.^78^. We correlated blood and fibroblast eQTL data to check whether the use of blood data with large sample sizes for discovery, then fibroblast data for validation, was justified.

#### Cell population-relevant genes

Next, we estimated enrichment of genes implicated by genetic risk loci in DD cell populations, since genes expressed in relatively few cell types are hypothesized to affect cell type functions. We studied single cell RNA sequence (scRNAseq) data from nodules of six DD patients containing seven cellular subtypes^60^. Using SNPsea, we prioritized cell types that are specific for Dupuytren’s disease. We mapped the associated loci to candidate genes, calculated specificity scores of these genes to cell populations, and tested their significance^79^. We used default settings, except for ‘–slop’ (1 × 10^5^), ‘–min-observations’ (100), and ‘–max-iterations’ (1 × 10^6^). A heatmap showing specificity scores of significant meta-GWAS loci in cell populations was created with the R-package heatmaply (version 1.3.0)^80^. For clustering we used heatmaply’s default Euclidean distance measure and the average linkage function^80^.

### Polygenic risk score calculation

We calculated polygenic risk scores (PRS) for the two Dutch and two UK cohorts in order to study the variance explained (i.e. narrow-sense heritability) by genetic risk variants identified in the meta-GWAS. First, we re-ran the meta-analysis of GWASs with the same settings as described above, using a leave-one-out approach to acquire summary statistics that are independent of said cohort and could thus be used for PRS calculation. Then, we used SBayesRC (v.0.2.0) to improve polygenic risk prediction by integrating effect sizes from our summary statistics with functional genomic annotations^81^. Next, we constructed PRSs with PLINK using the improved weights calculated by SBayesRC^65^. Logistic regression analysis was performed to associate the PRS with the outcome DD adjusting for age, sex and principal components (PCs). Last, we calculated liability-adjusted Nagelkerke’s pseudo *R*^2^ measures to scale the phenotypic variance explained by PRS to the (previously estimated) disease prevalence of 7.08% (Dutch population) and 13.40% (UK population) that has been corrected for the general population of all ages, instead of the disease prevalence in the study population which was affected by ascertainment bias^82,83^.

### Genetic correlations

We assessed shared genetic etiology between DD and twelve clinically associated traits: body mass index (BMI), high-density lipoprotein (HDL), triglycerides, type 2 diabetes mellitus (T2D) adjusted for BMI, T2D unadjusted for BMI, T2D in European UK Biobank subjects with only variants available from the Haplotype Reference Consortium, fasting glucose, HbA1c, idiopathic pulmonary fibrosis, systemic sclerosis, frozen shoulder, psoriasis, bone mineral density, and height^20,21,55,64,84–93^. We employed LD score regression to calculate genetic correlations, using full GWAS summary statistics of DD and traits of interest^94^. We assessed the significance of the genetic correlation with a Bonferroni‐corrected threshold being α = 0.05/14 = 0.0036.

### Colocalization analysis

For traits sharing a significant genetic correlation, we first selected genetic loci significantly associated to both traits. Then, SNPs from a 200 kb block surrounding the significantly associated SNPs were selected. Colocalization analysis was performed with the coloc and the Sum of Single Effects (SuSie) R-packages^95^, using data from the 1000 Genomes Phase 3 dataset from the European continental population (version v5a, Feb. 20^th^ 2015)^62^, and calculated raw inter-variant allele count correlations (–r) with PLINK^65^. Missing correlation values were set to zero. Colocalization analysis estimates the posterior probability of a shared causal variant, a high posterior probability value indicating a shared variant.

### Data visualization

A Manhattan plot was created with the GWASinspector package (version 1.5.1)^67^. Gene-based analysis (as computed by MAGMA) and gene Manhattan plot were performed and visualized with FUMA (v1.3.6) SNP-to-Gene function^70,96^. Forest plots of the odds ratios and confidence intervals of all meta-GWAS hits were constructed in R (version 3.6.1) using the rmeta package (https://CRAN.R-project.org/package = rmeta, version 3.0). Regional association plots of each SNP were created with Locuszoom (version 0.13.2)^97^.

### Reporting summary

Further information on research design is available in the Nature Portfolio Reporting Summary linked to this article.

### Supplementary information

Supplementary Information

Description of Additional Supplementary Files

Supplementary Data 1

Supplementary Data 2

Supplementary Data 3

Supplementary Data 4

Supplementary Data 5

Supplementary Data 6

Supplementary Data 7

Supplementary Data 8

Supplementary Data 9

Supplementary Data 10

Supplementary Data 11

Supplementary Data 12

Supplementary Data 13

Supplementary Data 14

Supplementary Data 15

Supplementary Data 16

Supplementary Data 17

Supplementary Data 18

Supplementary Data 19

Supplementary Data 20

Supplementary Data 21

Supplementary Data 22

Supplementary Data 23

Supplementary Data 24

Reporting Summary

## Data Availability

The summary statistics data generated in this study have been deposited in the GWAS catalog under accession code GCST90301252. The raw genotype data are protected and are not available due to data privacy laws. The open source data used in this study from FinnGen (v6) [https://r6.finngen.fi/pheno/M13_DUPUTRYEN], 1000Genomes (phase 1 and 3) [https://www.internationalgenome.org/data/], and the Haplotype Reference Consortium [http://www.haplotype-reference- consortium.org/] were downloaded from each of their respective websites. SNPs of interest were extracted with VCFtools [https://vcftools.github.io/]. GWAS Catalog database (version 21 April 2021) was used for in silico pleiotropy analysis. Single cell RNA sequencing data were aqcuired via correspondence. Blood cis-eQTL data were downloaded from the eQTLGen consortium [https://eqtlgen.org/]. Fibroblast cis-eQTL data were downloaded from the Genotype-Tissue Expression (GTEx) version 8 [https://gtexportal.org/home/]. Co-regulation analysis was performed using DEPICT and its accompanying expression dataset of 77,840 samples [https://github.com/perslab/depict]. The Phenoscanner database (version 2) was queried to look up quantitative trait loci (QTL) associations [http://www.phenoscanner.medschl.cam.ac.uk/login/?next = /data/]. GeneMANIA was used to construct composite networks of the prioritized genes based on the database accompanied by the software (build 12-02-2019) [http://genemania.org/]. The STRING database v11.0 to find the protein-protein interactions [https://version-11-0.string-db.org/]. The Genotype-Tissue Expression (GTEx) database (v8) was used to study gene expression of prioritized genes [https://gtexportal.org/home/]. To assess enrichment of tissue-specific genes, the Human Protein Atlas (PMID 25613900) and mouse gene expression as well as RNAseq data from the GTEx database were used [https://www.proteinatlas.org/].
