# Supplementary material for: A genome-wide association meta-analysis implicates Hedgehog and Notch signaling in Dupuytren’s disease
Source: Nat Commun. 2024 Jan 3;15:199. doi: 10.1038/s41467-023-44451-0 (PMC10764787; doi:10.1038/s41467-023-44451-0)
Supplement: Supplementary file 1 — Supplementary Information [file 41467_2023_44451_MOESM1_ESM.pdf]

**Supplementary information – A genome-wide association meta-analysis implicates Hedgehog and Notch signaling in Dupuytren's disease**

**SUPPLEMENTARY METHODS**

## Genotype quality control procedures

### The Lifelines cohort description

Lifelines is a multi-disciplinary prospective population-based cohort study examining, in a unique three-generation design, the health and health-related behaviors of 167,729 persons living in the North of the Netherlands. It employs a broad range of investigative procedures in assessing the biomedical, socio-demographic, behavioral, physical and psychological factors which contribute to the health and disease of the general population, with a special focus on multi-morbidity and complex genetics.

### Quality control procedures of Lifelines control data

A detailed description of the Lifelines cohort genotype calling and quality control (QC) pipeline can be found on Github (https://github.com/molgenis/GAP). In brief, DNA samples were genotyped with the Illumina HumanCytoSNP-12 (CytoSNP) array and the Illumina Global Screening (GSA) array, and called with GenomeStudio and OptiCall, respectively.(1) QC was performed with PLINK.(2) In the first QC, a low cutoff call rate of 80% was used to remove both low quality samples and markers. Next, a more stringent cutoff of 99% was used both for samples and markers. Monomorphic markers (minor allele frequency [MAF]=0) and markers with a low p-value ($<1x10^{-6}$) for deviations from Hardy-Weinberg equilibrium (HWE) were excluded. Samples were excluded if the sample heterozygosity (for the autosomal markers) deviated more than four standard deviations from the expected mean conditional on runs of homozygosity, as high heterozygosity indicates potential DNA contamination. We checked sex discrepancies between recorded sex of individuals in the database and sex based on X chromosomes homo-/heterozygosity and updated sex according to genotype data, if a sample switch was detected. Otherwise, the sample was excluded. Next we removed duplicate and related samples (identity by descent [IBD]) when the relatedness did not match with that mentioned in the databases after additionally checking for sample swaps. We used PLINK's 'genome' function to calculate IBD with the criteria a pi-hat (average IBD sharing) of >0.99 for duplicates, between 0.35 and 0.99 and between 0.15 and 0.35 for first- and second-degree relatives, respectively, and <0.05 for unrelated individuals.(2) To check genetic ancestry, non-HLA genotype data were merged with a dataset of 1000Genomes Phase 3 samples containing variants with a MAF>5%.(3) This merged dataset was pruned ($r^2>0.1$) and principal components (PCs) were calculated with PLINK.(2) Non-European samples based on the first two PCs (>7SD from mean of 1000Genomes European samples) were removed.

For this study, all first- and second-degree related samples were removed to obtain a set of independent individuals, since the Lifelines cohorts were much larger than the Dupuytren case cohorts and a control sample size that is more than four-fold larger than that of the cases does not yield more statistical power. Genome-wide complex trait analysis (GCTA) was used to determine a set of unrelated Lifelines controls for both the CytoSNP and GSA array separately.(4) Next PLINK was used to determine the relatedness between the Lifelines cohorts.(2) We removed duplicates and first- or second-degree relatives from the CytoSNP release, since the GSA chip contains more genetic variants (~650,000 vs ~300,000).

### Quality control procedures of Dupuytren cases

QC was performed for each Dupuytren release separately (CytoSNP and GSA), using PLINK (version 1.9) and R (version 3.6.1).(2,5)

Preparations

The positions of the CytoSNP data were remapped from build 36 to build 37 (GRCh37, hg19) using liftOver (http://hgdownload.cse.ucsc.edu/admin/exe/linux.x86_64/). All markers were aligned and GSA SNP ids were converted to rs-ids according to the Illumina GSA manifest. In addition, multiple variants on the same position were harmonized: if for duplicate variants the alleles did not match, while being the same type (i.e. both SNPs, or both insertion/deletion polymorphisms) or genotype concordance was low (>100 differences), the variants were removed from the dataset. If the genotype concordance was high, the data of the multiple entries was merged maximizing genotype information (i.e. overwriting a missing genotype at the first entry with that of the second). SNPs from chromosome X and Y, and mitochondrial SNPs were removed.

Genotype calling and QC of the CytoSNP cases and GSA cases

Dupuytren cases and the Lifelines controls were genotyped separately as they originate from two separate studies. To reduce batch effects for the GSA genotyping data as much as possible, we combined the raw data of probe intensities of all cases and 1200 random controls and called genotypes together using optiCall.(1) For the CytoSNP data, calling was done separately, because the raw data were not available any more. We applied the QC pipelines of the respective control cohorts to QC our case cohorts, adapting them for cases where necessary. That is, as a first step for the case cohort QC we extracted only variants that survived QC from the Lifelines control cohort from the respective genotyping platform. Furthermore, the HWE p-value threshold was released to $1x10^{-10}$, since in cases there may be deviation due to the disease model. Lastly, a QC step was added in which

the allele frequencies of cases are compared to those of controls and genetic markers with an allele frequency that deviated between cases and controls with a chi-square p-value <1x10$^{-6}$ were removed from the case dataset. We hypothesized that this significance was more likely due to genotyping assay failures than true causality and expected true causal hits to be retrieved during imputation.

**Removal of related individuals**
As the case and control datasets both originated from the same geographical region, overlap and relatedness between these datasets was plausible. Therefore, we calculated relatedness with PLINK (pi-hat≥0.15) to estimate genetic relationships between the four cohorts (cases or controls from CytoSNP or GSA).(2) If Dupuytren cases or their relatives also participated in the control cohort, they were removed from the control cohort.

**Genotype imputation**
Imputation was performed for each Dupuytren and Lifelines release separately (CytoSNP and GSA). After QC, both datasets were converted to Variant Call Format (VCF) separately and uploaded to the Sanger Imputation Server (https://www.sanger.ac.uk/tool/sanger-imputation-service/). Imputation was next done using the 1000Genomes phase 1 for the CytoSNP cohorts and the Haplotype Reference Consortium as reference panel for the GSA cohorts to match the Lifelines Cohort QC pipelines.(3,6,7)

**Merging of case and control datasets**
As the mean age of DD cases was higher than that of controls, only controls with an age range similar to DD (mean 62 years, IQR 56-70 years) were selected. Imputed genotype data of cases and controls were merged per chromosome for each genotyping release using BCFtools (v1.16).(8) Alleles were flipped and SNP identifiers were converted to a chromosome-position-reference allele-alternative allele format, in order to keep multiallelic variants. (9)Ten PCs were calculated with PLINK using the merged, pruned genotyped data of the DD cases and Lifelines controls for both genotyping platforms, separately, to correct for population stratification in the association analyses.(2)

**GWAS QC**
Imputation quality (info scores) and MAF thresholds were set for each cohort. For the Dutch CytoSNP cohort, the MAF threshold was 0.03, the maximum MAF difference between cases and controls was 0.05, and the imputation quality threshold was 0.8. For the Dutch GSA cohort, the MAF threshold was 0.01 and the imputation quality threshold was 0.8. For the UK BSSH-GODD, UK Biobank, German Affymetrix SNP, and German Affymetrix Axiom cohorts the MAF threshold was 0.01 and the imputation quality threshold was 0.3.

**Bioinformatic follow-up analyses**

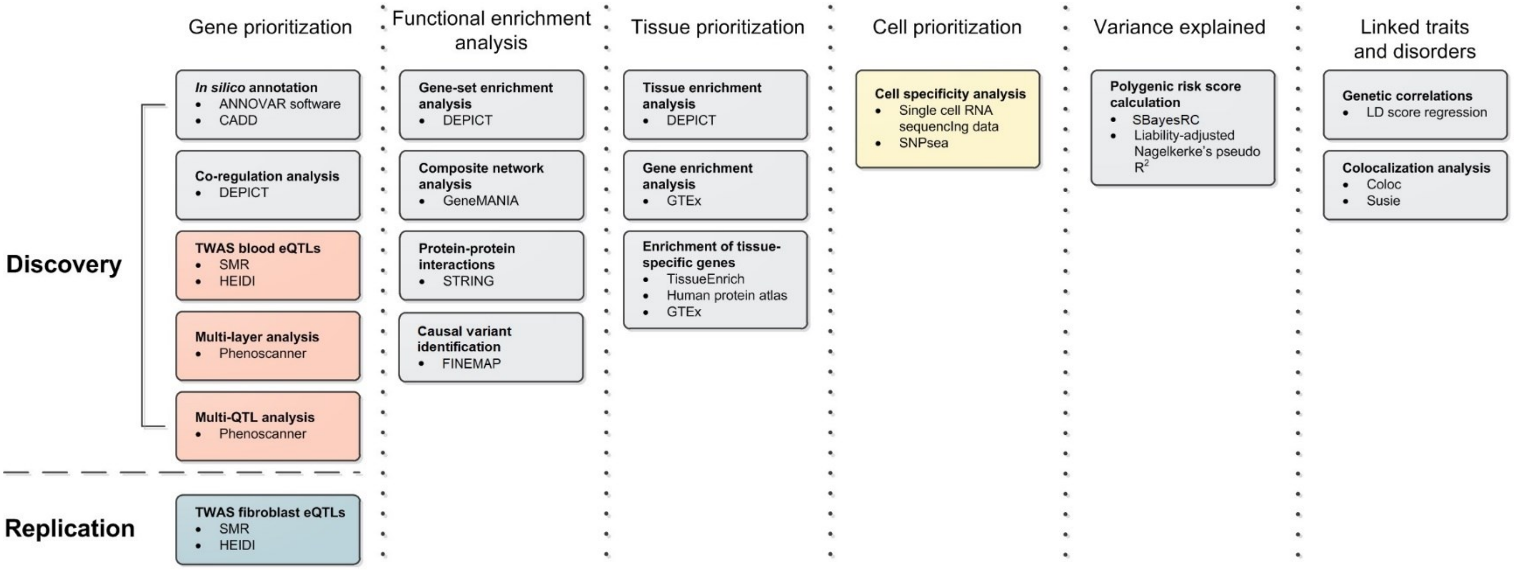


**Supplementary Figure 1.** Overview of follow-up analyses after meta-GWAS. Grey boxes detail bioinformatic approaches not using eQTL data. Red boxes detail analyses using blood eQTL data. The blue box details an analysis using fibroblast eQTL data. The yellow box details an analysis using single cell sequencing data from Dupuytren's nodules.

## Co-regulation analysis

Functionally similar genes residing at different loci that are genome-wide significantly associated to a trait or disease of interest are hypothesized to have a higher probability to be causally involved, likely via acting through shared mechanisms. In order to identify functionally similar genes within DD associated genomic loci, co-regulation analysis was performed, using DEPICT(10) and its accompanying expression dataset of 77,840 samples. We used default settings: a p-value threshold of $5\text{x}10^{-8}$, an $r^2$ of 0.1 (as an LD metric), and a physical distance of 500 kb for clumping. To extract genes, locus boundaries of $r^2$>0.5 were set on either side of independent hits. DEPICT was run over the full set of DD meta-GWAS summary statistics.

## Transcriptome-wide association study (TWAS)

Blood data

In order to identify genes whose expression levels are truly associated with DD free of non-genetic confounders, Summary-data-based Mendelian Randomization (SMR) analysis was performed (11), integrating the DD meta-GWAS results with gene expression data. We used the blood cis-eQTL data from the eQTLGen (n~32,000) consortium (12). Genotype data from the European continent population of the 1000 Genomes Project Phase3 version 5a (3) were used for linkage disequilibrium (LD) calculations. Variants with inconsistent alleles or allele frequency differences >0.2 amongst pairs of the three input datasets (i.e., eQTL, LD reference, and meta-GWAS dataset) as well as variants within the MHC region were excluded from the analysis. Since the presence of LD between distinct eQTLs and meta-GWAS SNPs may cause spurious SMR associations, we used the heterogeneity in dependent instruments (HEIDI) test (11) to filter out the possibly confounded SMR significant results. Because 15,491 genes were tested, a Bonferroni corrected significance level of <$3.23\text{x}10^{-6}$ (i.e. 0.05/15,491) was used for SMR, and a level of ≥$2.08\text{x}10^{-3}$ (i.e., 0.05/number of SMR significant genes) for the HEIDI test.

Fibroblast data

For SMR analysis with fibroblast data, we used fibroblast cis-eQTL data from the Genotype-Tissue Expression (GTEx) version 8 (n=483) consortium(13) and the European continent population of the 1000 Genomes Project Phase 3(3) version 5a data for linkage disequilibrium (LD) calculations. To match ancestry with our GWAS data, we used the cis-eQTL mapping results for the European subset of GTEx donors. To avoid bias in p-values when re-calculated by SMR software, we adjusted standard errors as SE=b/z* with z* being computed based on the original p-values and b being the effect size. We used the heterogeneity in dependent instruments (HEIDI) test (11) to filter out the significant SMR results that were potentially confounded by the presence of LD between distinct eQTL and GWAS SNPs. Variants with inconsistent alleles or allele frequency differences >0.2 amongst pairs of the three input datasets as well as variants within the MHC region were excluded from the analysis. To identify new significant genes, the following criteria was applied: SMR p-value <$6.84\text{x}10^{-6}$ (Bonferroni corrected significance level considering 7,307 genes being tested), and a HEIDI p-value of ≥$7.14\text{x}10^{-3}$ (i.e., $0.05/n_{sig}$; with $n_{sig}$ as the number of SMR significant

genes). SMR analysis in fibroblasts was also used to examine the contribution of the prioritized genes from other gene-prioritization analyses to DD in a disease-relevant tissue.

**Multi-layer analysis**

Targeting genes with multi-layer molecular associations (ML genes; Supplementary Figure 7) as well as genes being regulated by DD meta-GWAS loci with evidence from more than one molecular layer (MultiQTL genes; Supplementary Figure 8), a multi-layer analysis of all significant genomic loci from the meta-GWAS was performed. We used the *in silico* sequencing results (see Methods paragraph '*In silico* annotation' in main text) with an $r^2$ threshold of 0.8 and excluded variants within the MHC region. Next the Phenoscanner(14) database (version 2) was queried to look up quantitative trait loci (QTL) associations across different molecular layers including DNA methylation, gene expression, protein, and metabolite levels. A physical distance threshold of 100 kb was set for mapping DNA methylation probes to their nearest genes based on Ensembl GRCh37 release 104. Genes with variants affecting three or four molecular layers were flagged as ML genes and downstream regulated genes with support from more than one molecular layer as MultiQTL genes.

**Identifying causal variants**

FINEMAP was used to identify the most likely causal variants of the meta-GWAS.(15) We started with the list of 56 index SNPs, their linked SNPs ($r^2$>0.5), and all other genome-wide significant SNPs in the region. The MHC locus, i.e., rs886423, was excluded owing to its complex LD structure. Then, based on the above list, we constructed 55 z-files as genomic regions providing info for the contained SNPs. Next, LD matrices were constructed for these SNPs based on 1000G phase 3 reference panel of European individuals, using PLINK 2.0.(16) Finally, we performed FINEMAP v1.4.2 on the 55 genomic regions with default settings to identify most likely causal SNPs in each genomic region.(15)

**Functional enrichment analysis**

<u>DEPICT</u>

In order to predict pathways involving genes within the identified Dupuytren's GWAS loci, we conducted gene-set enrichment analysis using DEPICT.(10) This approach enables functional predictions to also account for uncharacterized genes according to co-expression data. Our analysis was based on the same settings as for DEPICT gene prioritization (see Supplementary Methods paragraph 'co-regulation analysis'). We ran DEPICT over the full set of GWAS summary statistics of Dupuytren's disease.

<u>GeneMANIA</u>

First, we merged the prioritized gene lists from the previous steps, i.e., 1) genes with non-synonymous variations linked to Dupuytren's GWAS loci (n=7), 2) genes with their expression levels associated with Dupuytren's disease (n=8), 3) co-regulated genes within Dupuytren's loci (n=27), 4) genes with multi-layer molecular associations (n=23), and 5) downstream genes with multiple QTL associations for Dupuytren's loci (n=84). After removing duplicates and excluding MHC region, a list of 119 prioritized genes was obtained (Supplementary Data 17) and used for functional assessments. We also used the following subset gene lists for sensitivity analysis: 1) prioritized genes within Dupuytren's loci ($r^2$>0.5) (n=73), and 2) prioritized genes with more than one source of biological evidence (n=23).

Next, the GeneMANIA algorithm was used to construct composite networks of the prioritized genes based on the thorough database of different data types accompanied by the software (build 12-02-2019). In order to further enrich the networks, we added double the amount of genes of each prioritized gene list, selecting their top related genes (n=238, n=146, and n=46 respectively). Then performed gene ontology (GO) enrichment analysis provided by the GeneMANIA Cytoscape plugin.(17)

<u>STRING</u>

Directing towards proteome molecular layer, we used the STRING database v11.0 (18) to find the protein-protein interactions of our 119 prioritized genes. Functional enrichment analysis was performed based on the whole network. Only interactions with a high confidence (≥0.7) were studied and used to identify major connected components. We further sought for enriched functions through these subset networks.

**Tissue prioritization**

We investigated different bioinformatics approaches to find important tissues in which genetic factors of DD contribute in disease progression. First DEPICT analysis (10) was performed to find tissue/cell types in which genes from our DD loci ($r^2$ > 0.5) are highly expressed. This analysis was based on 209 tissue/cell types from ~37,000 human microarrays. The same settings were used as for DEPICT gene prioritization analysis (see Supplementary Methods section 'co-regulation analysis). Next, we examined the gene expression status (i.e., 0/1) of our 119 prioritized genes across 54 human tissues in the Genotype-Tissue Expression (GTEx) v8 database (13) and performed 10,000 permutations using random gene sets of the same count to see which tissues expressed our prioritized genes more than expected by chance. Finally, the TissueEnrich R package(19) (version 3.13) was used alongside with its processed data from the Human Protein Atlas (*PMID 25613900*) and mouse gene expression,(20) as well as

RNAseq data from the GTEx database (13) to assess enrichment of tissue-specific genes in our list of 119 prioritized genes. Tissue-specific genes were defined as genes with a minimum gene expression of 1 transcripts per million (TPM) that have at least five-fold higher expression in a certain tissue in comparison to all other tissues.

## SUPPLEMENTARY RESULTS

### Co-regulation analysis

DEPICT prioritized 27 genes from DD genomic loci with a functional similarity larger than expected by chance, designated by a false-discovery rate (FDR) <0.01 (Supplementary Data 18).

### Transcriptome-wide association study

The SMR analysis of blood eQTLs returned 24 genes of which expression levels were significantly associated with DD ($P_{SMR}<3.23x10^{-6}$). Eight of these genes also passed the heterogeneity test ($P_{HEIDI}\geq2.08x10^{-3}$). The expression levels of three genes, i.e., *CFDP1*, *MTOR*, and *PTPN4*, were positively correlated with DD; the other five genes (*CTD-2587M2.1*, *PJA2*, *TMEM98*, *AFAP1*, and *GFPT1*) were predicted to have protective effects (Supplementary Data 13).

### Multi-layer analysis of meta-GWAS loci

Multi-layer analysis of 85 genomic SNPs for Dupuytren's disease returned 139 SNPs, mostly intronic, mapping to 23 genes from 18 loci with multi-layer associations across three or four molecular layers. For two genes, variants were associated with all four molecular layers, i.e., *CFDP1* and *LOC101928748* (Supplementary Data 19, Supplementary Figure 9).

A total of 494 downstream genes was identified through association of DD loci with DNA methylation levels in the vicinity of genes (<100kb), gene expression levels, or protein levels. The associations of 84 of these genes was supported by more than one molecular layer (MultiQTL genes). *CNTN2* and *GDF15* were found in all three layers of DNA methylation, gene expression, and protein levels (Supplementary Data 20). Twenty-two out of the 84 MultiQTL genes were also shown among genes with ML associated variants, among the aforementioned *CFDP1* gene.

### Identifying causal variants

For almost all of the 55 index SNPs there was considerable evidence for being causal (posterior inclusion probability ~ 1 and log10 Bayes factor > 2, see Supplementary Data 8). As FINEMAP does not assume a single causal SNP, many loci have multiple likely causal SNPs.(15) The posterior estimated effect size ('mean_incl' column) helps selecting the most likely important SNP per locus. Out of the additional 30 index SNPs, only three (i.e., rs6977665, rs11200062, and rs11743708) showed considerable evidence for being causal. While intergenic variants showed higher average probability of being causal, the proportion of variants with a considerable evidence (e.g. log10 Bayes factor > 2) was the highest for exonic variants (Supplementary Figure 10).

### Functional enrichment analysis

<u>DEPICT</u>

DEPICT gene set enrichment analysis resulted in seven significant gene sets at an FDR<0.05 (Supplementary Data 9). Abnormal limb morphology was the term with the lowest p-value (p-value=$3.68x10^{-7}$). Other low-p-value terms include abnormal cartilage morphology, (p-value=$1.08x10^{-6}$), abnormal skeleton morphology (p-value=$3.57x10^{-6}$), as well as epithelial to mesenchymal transition (p-value=$1.95x10^{-6}$).

<u>GeneMANIA</u>

Ninety-seven out of the 119 prioritized genes could be identified by GeneMANIA, for which the functional enrichment analysis resulted in 379 significant terms (Supplementary Data 10). Positive regulation of cell migration was the most significant pathway (q-value=$3.34x10^{-12}$) involving 29 out of 97 identified prioritized genes (~30%). Other significant terms include stress-activated MAPK cascade, neuron projection guidance, extracellular matrix organization and cell-matrix adhesion, skeletal system and endoderm morphogenesis, and a number of related terms. Sensitivity analysis results were supportive by ~84% and 74% similarity with the original analysis (Supplementary Data 21 and 22).

<u>STRING</u>

We found 37 highly confident (≥0.7) interactions among the prioritized genes' products, which was significantly more than expected by chance (p-value=$9.74x10^{-5}$) (Supplementary Figure 11). Considering the ratio of observed to expected genes, sclerotome development was the most strongly enriched term. The full list of enriched pathways is represented in Supplementary Data 23. Two major connected components were identified in the network (Supplementary Figure 12). The first one including the *UBA52* gene as the central node, was enriched in response to stress as the most significant term after a number of general processes (Supplementary Data 24). The second connected component including the *TNC* gene was enriched in among others bacterial invasion of epithelial cells, human papillomavirus infection, and extracellular matrix organization (Supplementary Data 24).

### Tissue prioritization

Tissue enrichment analysis using DEPICT returned arteries as tissues in which genes within DD loci are highly expressed (FDR < 0.01, Supplementary Figure 5). Muscles, chondrocytes, and cartilage were also among the top results (FDR<0.2). Gene

expression status of our 119 prioritized genes across 54 human tissues from GTEx v8 database suggested muscular tissues, fibroblasts, blood, liver, and brain regions to significantly express DD prioritized genes (FDR<0.05, Supplementary Data 24, Supplementary Figure 13). Enrichment of tissue-specific genes across our list of prioritized genes returned muscles and arteries as the top findings (Supplementary Figure 14).

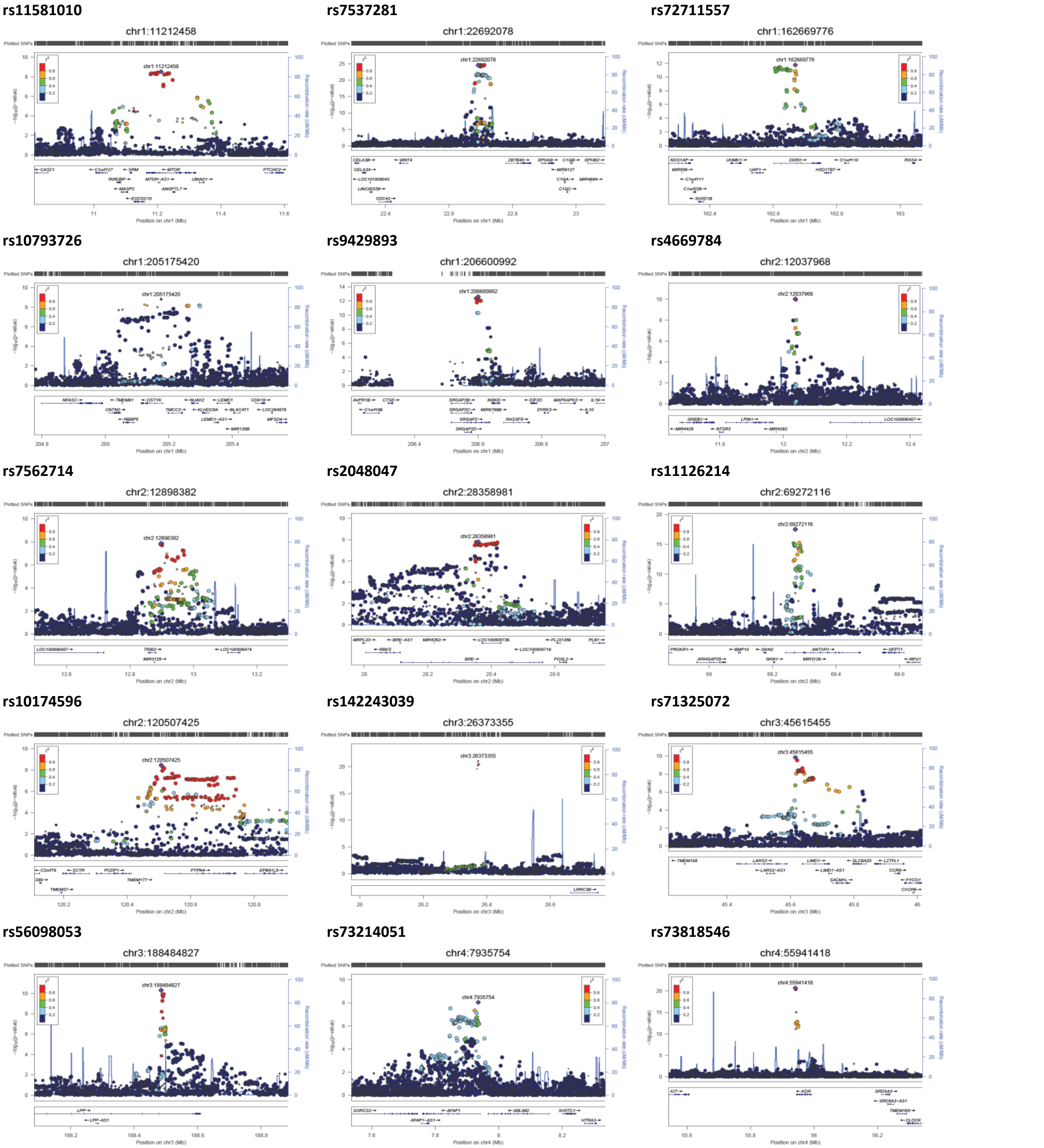


**Supplementary Figure 2.** Regional association plots of the 85 significantly associated independent DD SNPs (generated with LocusZoom). Lead SNPs are indicated with a purple diamond. In the plots chromosome and position are given for the lead SNPs. The color of the dots indicates strength of LD ($r^2$) with the lead SNP. The y-axis on the left represents the $-\log_{10}$(p-value) of genome-wide association. The x-axis depicts the position on the chromosome (in Mb). Known genes are listed above the x-axis. Symbols for SNP annotation are: framestop (triangle), splice (triangle), non-synonymous (inverted triangle), synonymous (square), UTR [untranslated region] (square), TFBScons [in a conserved region predicted to be a transcription factor binding site] (eight point star), MCS44 [in a region highly conserved within placental mammals] (square with two diagonal lines) and non-of-the-above (filled circle). The size of the SNPs indicates the sample size they were represented in.

rs1965670

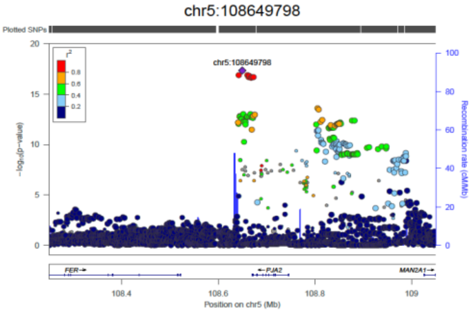


rs74516628

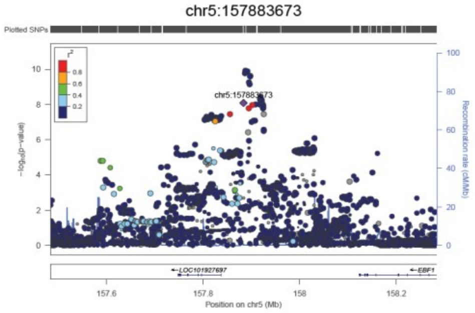


rs11743708

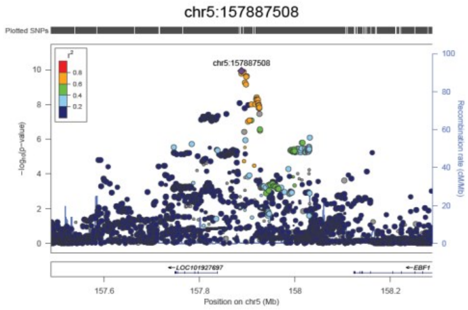


rs873560

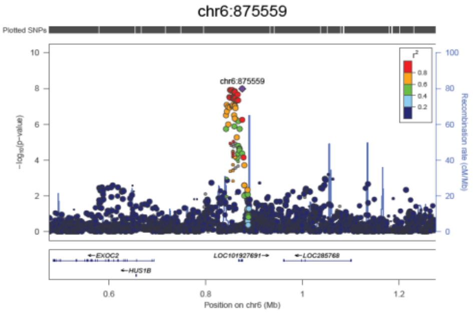


rs886423

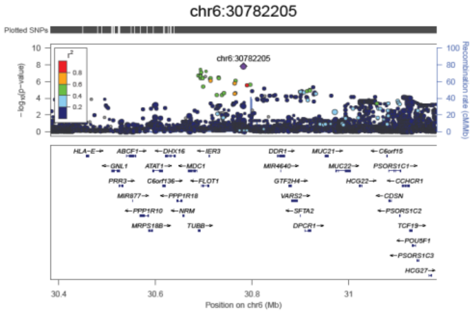


rs34203781

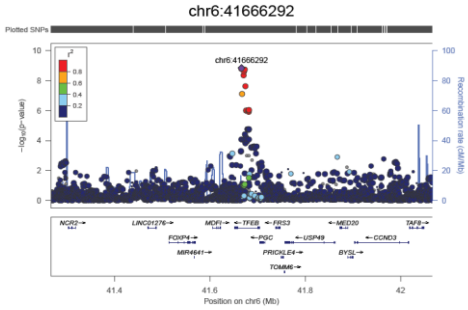


rs11966122

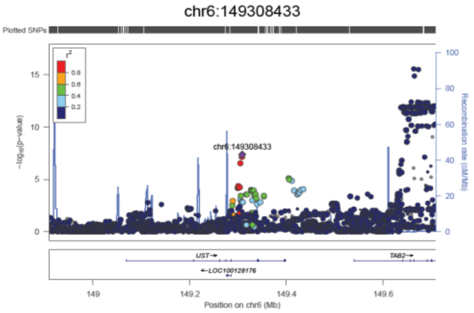


rs366905

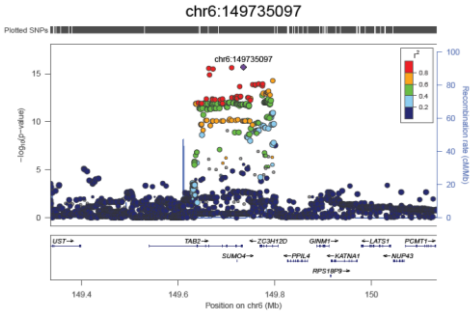


rs9770861

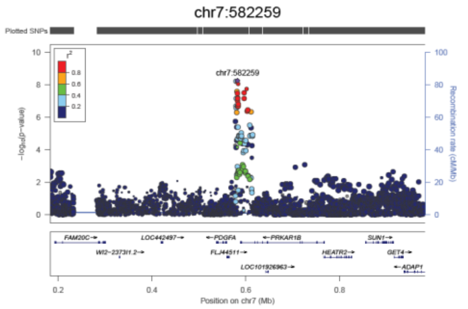


rs10264803

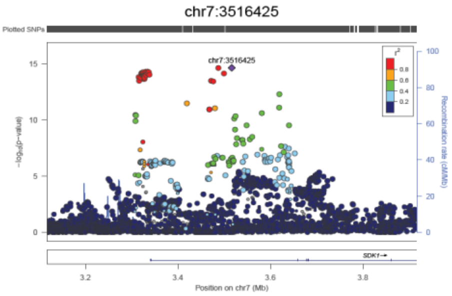


rs6462772

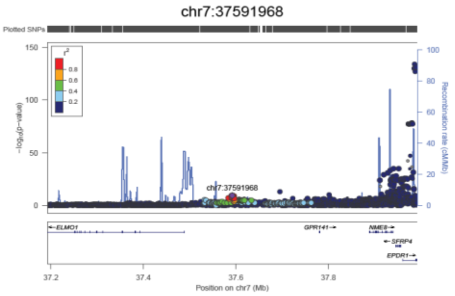


rs149796768

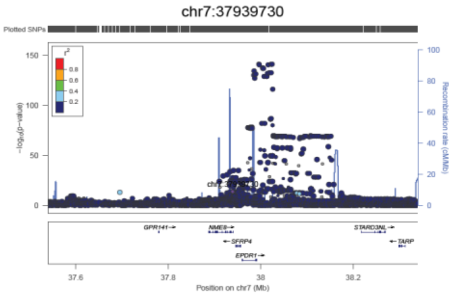


rs2598100

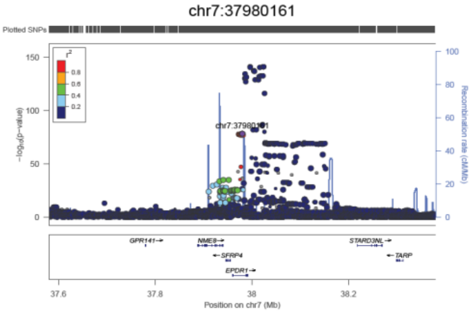


rs62443137

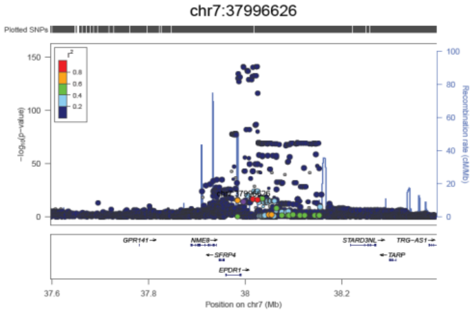


rs2044830

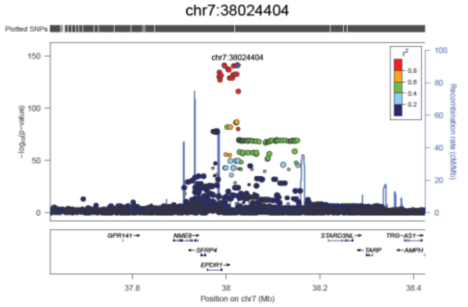


**Supplementary Figure 2** (continued)

**rs75958946**

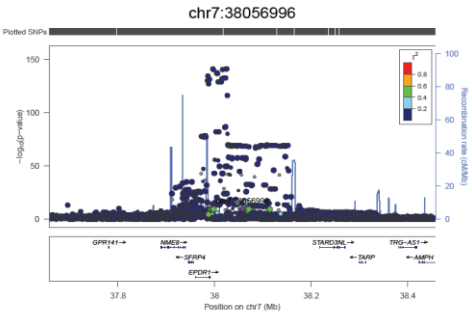


**rs75306755**

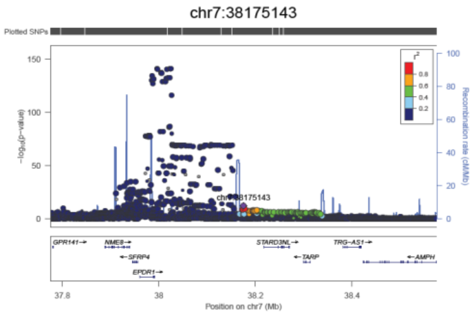


**rs38896**

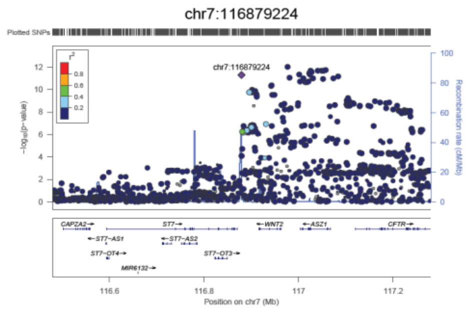


**rs6977665**

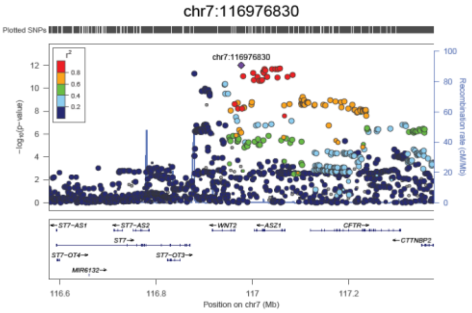


**rs17238923**

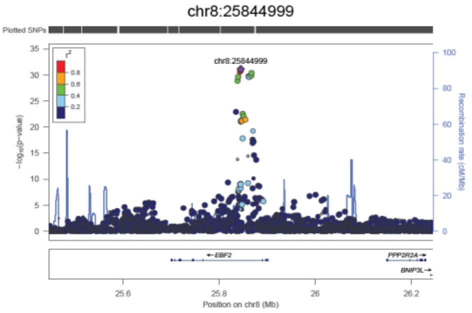


**rs6472419**

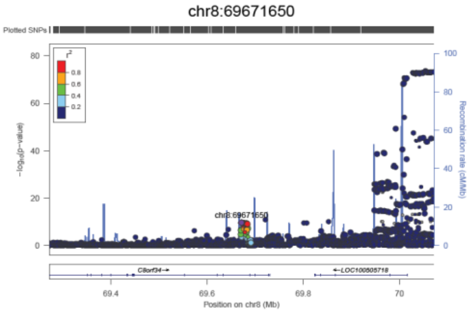


**rs2162353**

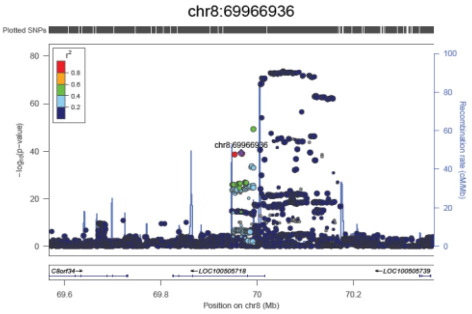


**rs2472141**

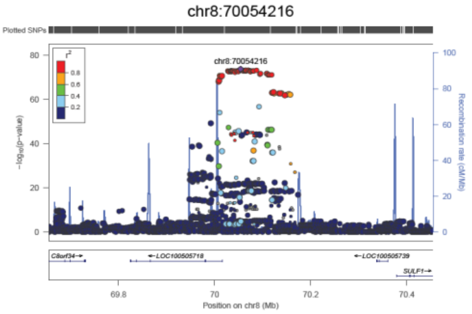


**rs10100769**

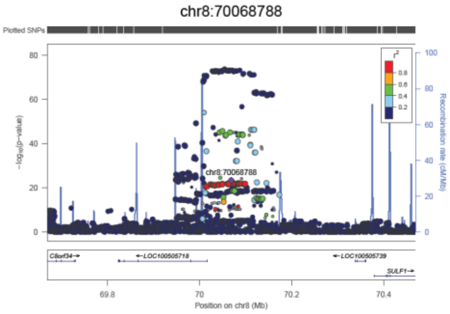


**rs13439053**

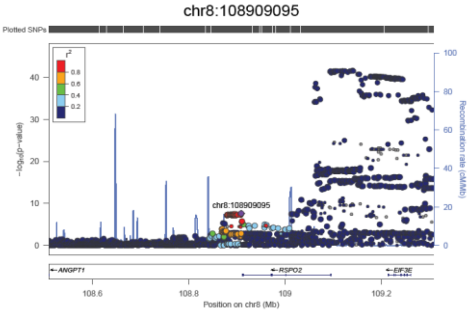


**rs648596**

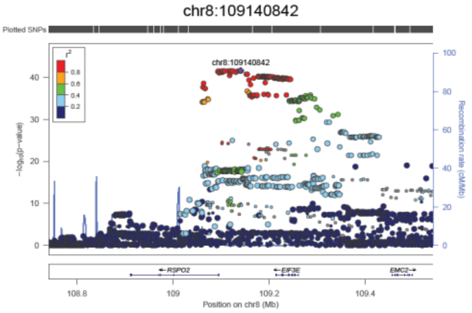


**rs10089785**

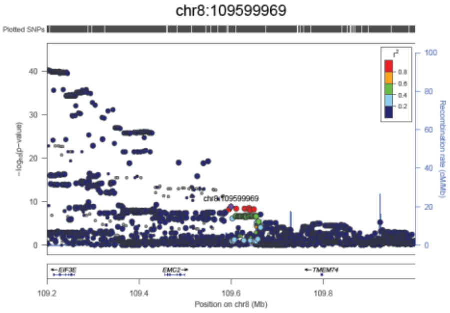


**rs62524109**

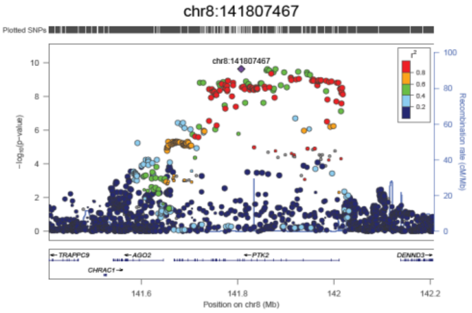


**rs7816361**

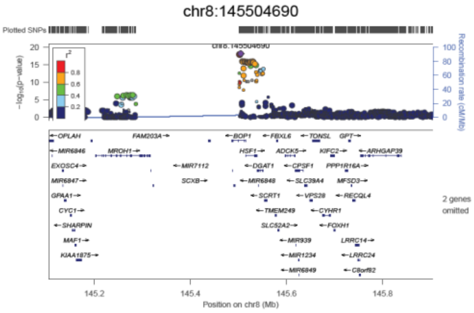


**rs12353046**

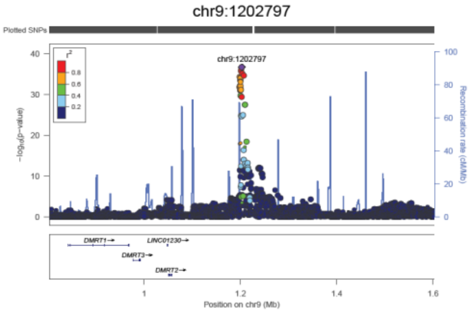


**Supplementary Figure 2** (continued)

rs56381416

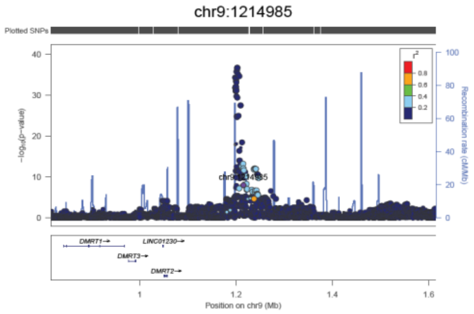


rs34810955

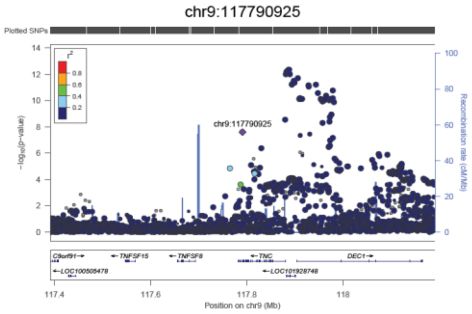


rs10125663

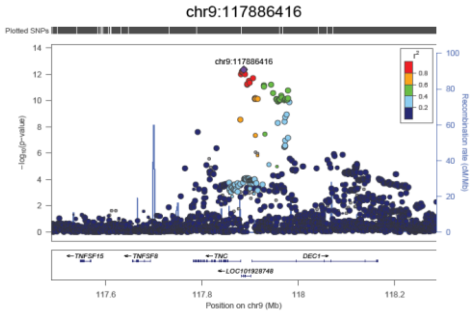


rs1649199

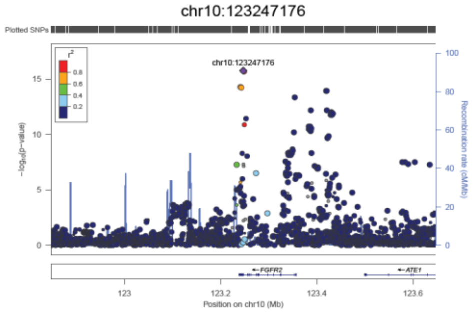


rs11200062

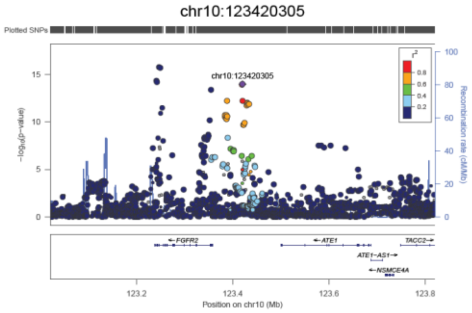


rs10831757

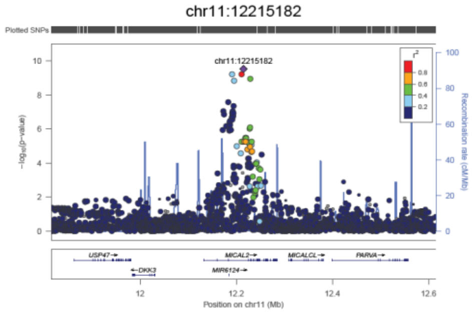


rs7307913

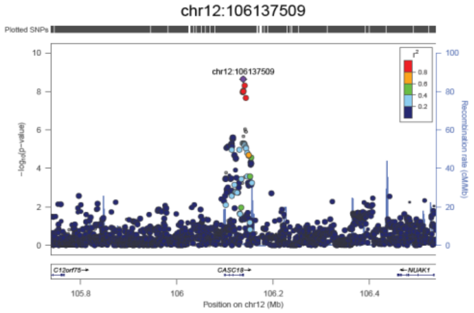


rs77660995

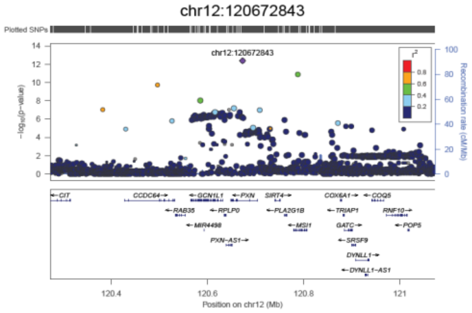


rs4942308

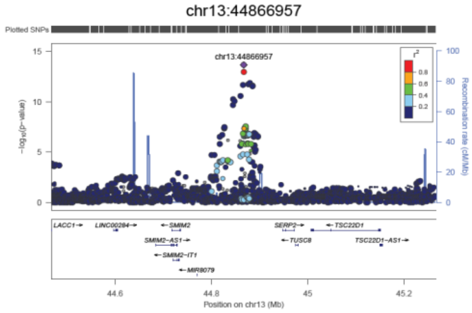


rs1042704

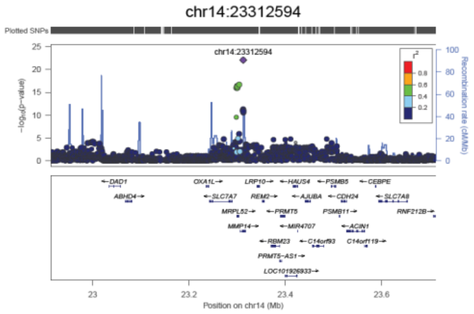


rs12881869

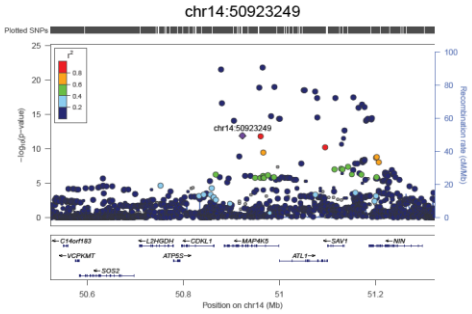


rs17791680

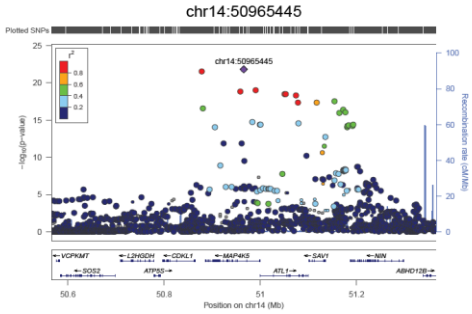


rs8032158

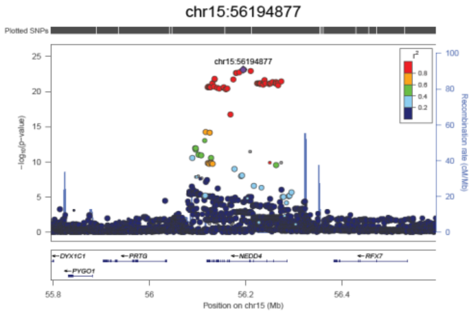


rs34412930

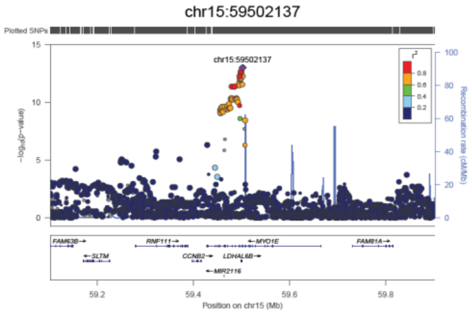


rs28522005

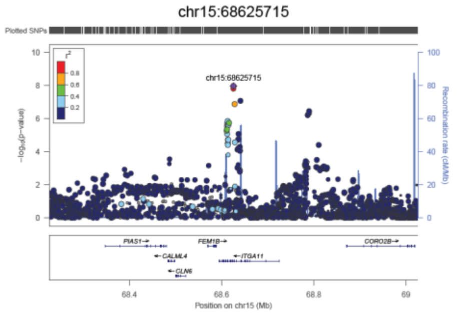


**Supplementary Figure 2** (continued)

rs12441312

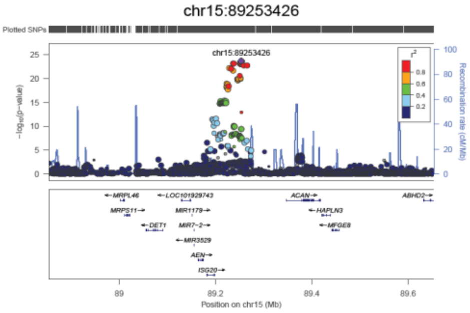


rs12442366

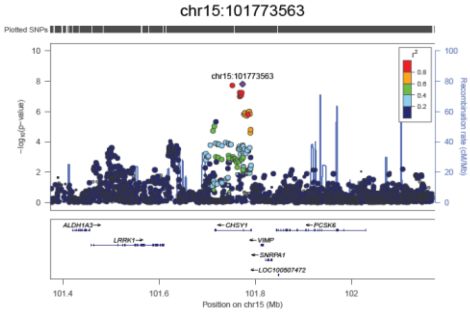


rs247436

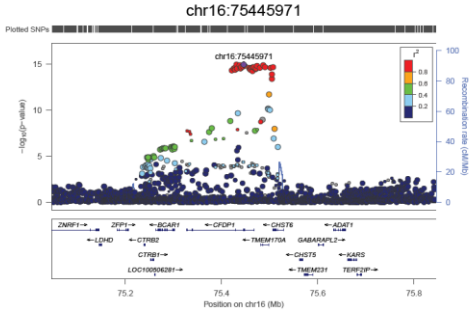


rs61092548

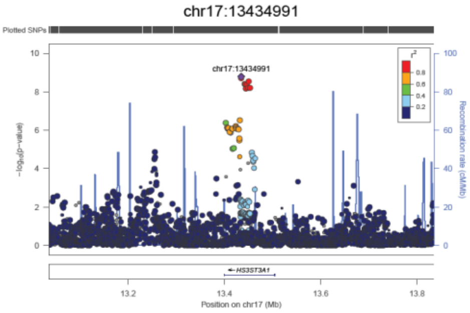


rs77727254

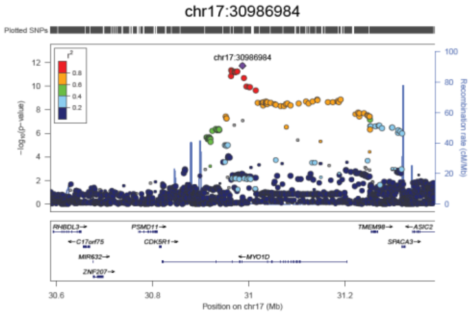


rs1398882

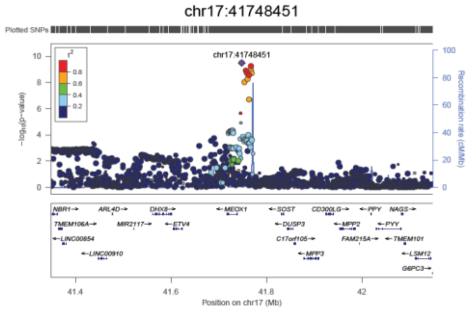


rs11665156

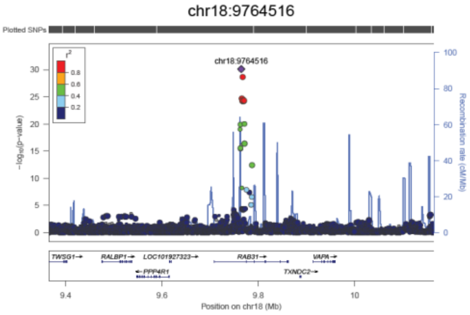


rs78030362

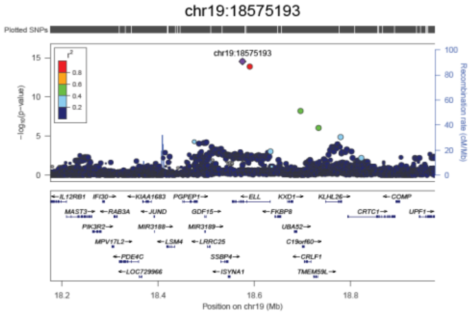


rs11672486

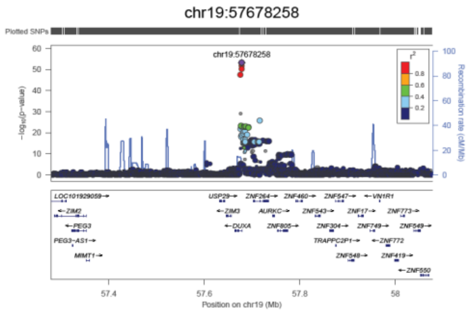


rs16988531

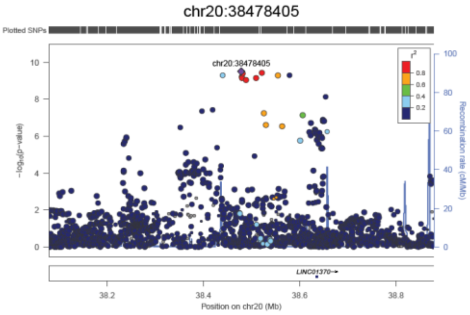


rs57082097

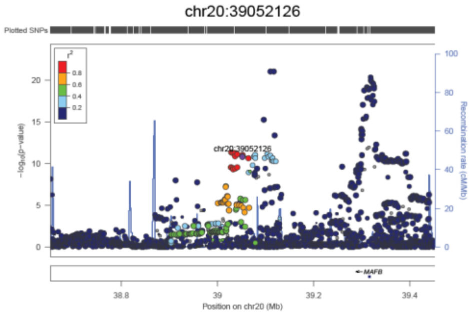


rs12481661

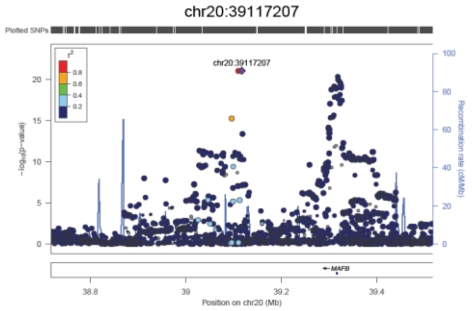


rs181089987

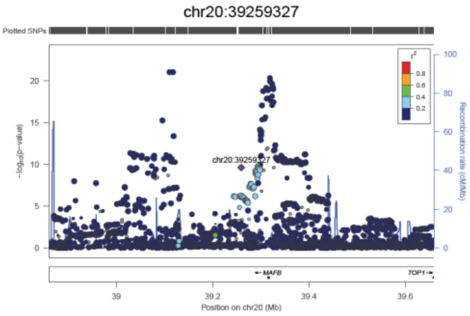


rs58716951

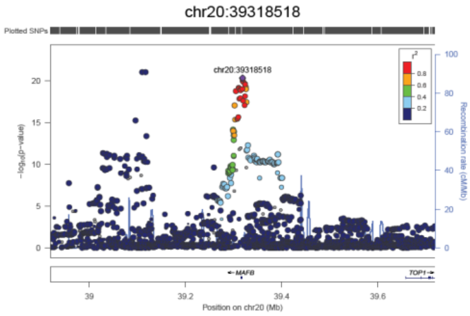


rs73130881

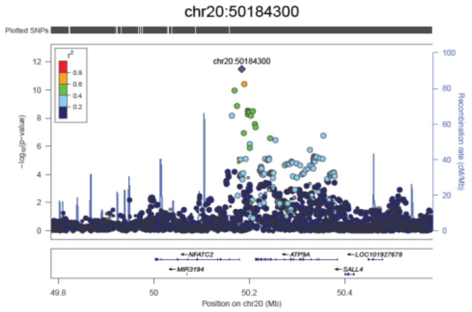


**Supplementary Figure 2** (continued)

**rs235931**

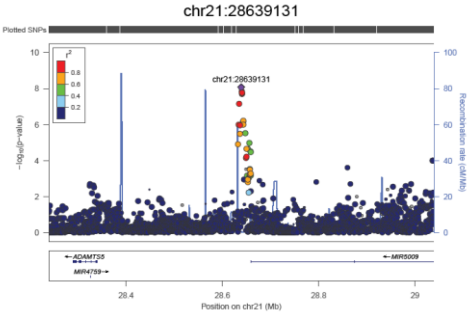


**rs117999064**

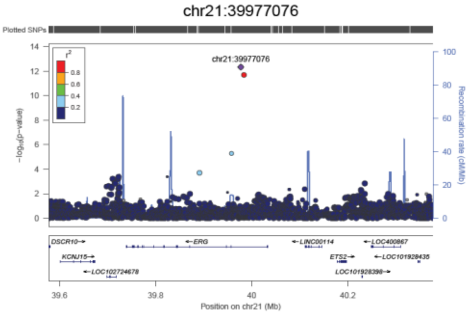


**rs17573837**

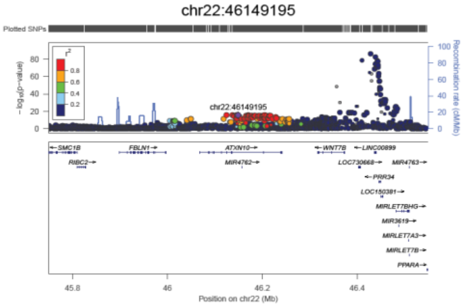


**rs11704955**

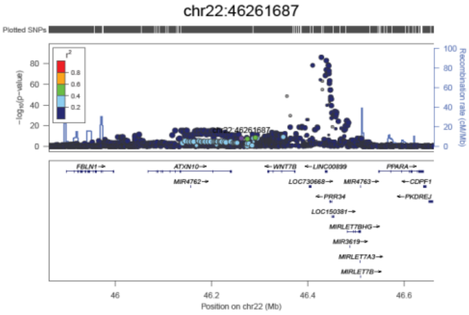


**rs28363932**

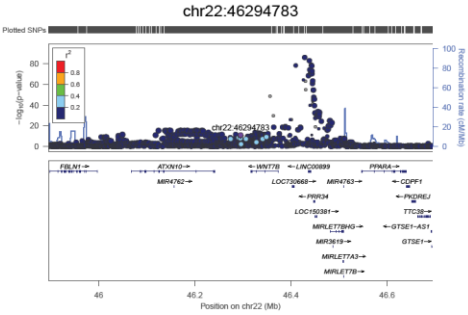


**rs28755830**

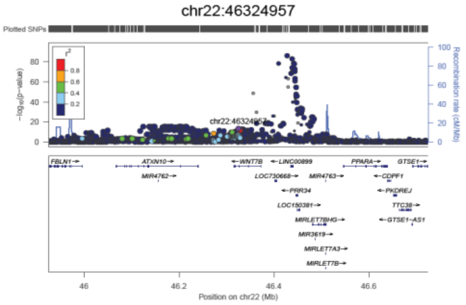


**rs145039496**

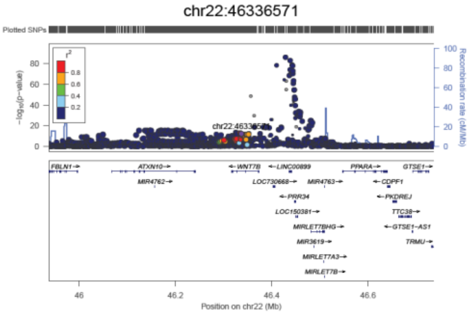


**rs117707689**

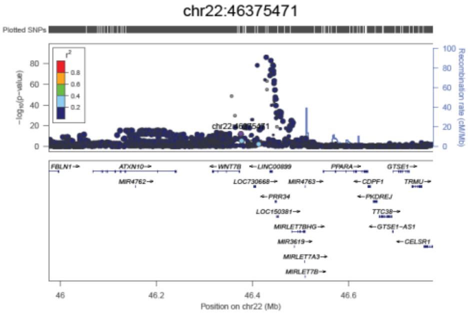


**rs112116858**

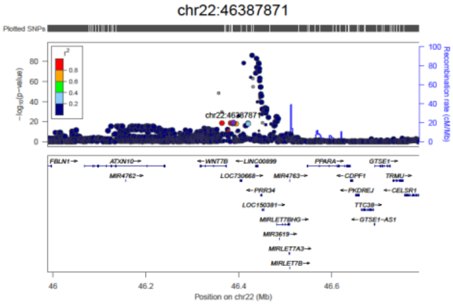


**rs9626908**

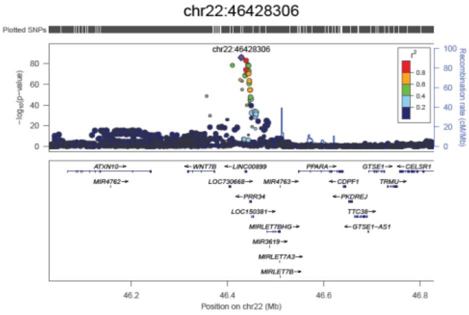


**Supplementary Figure 2** (continued)

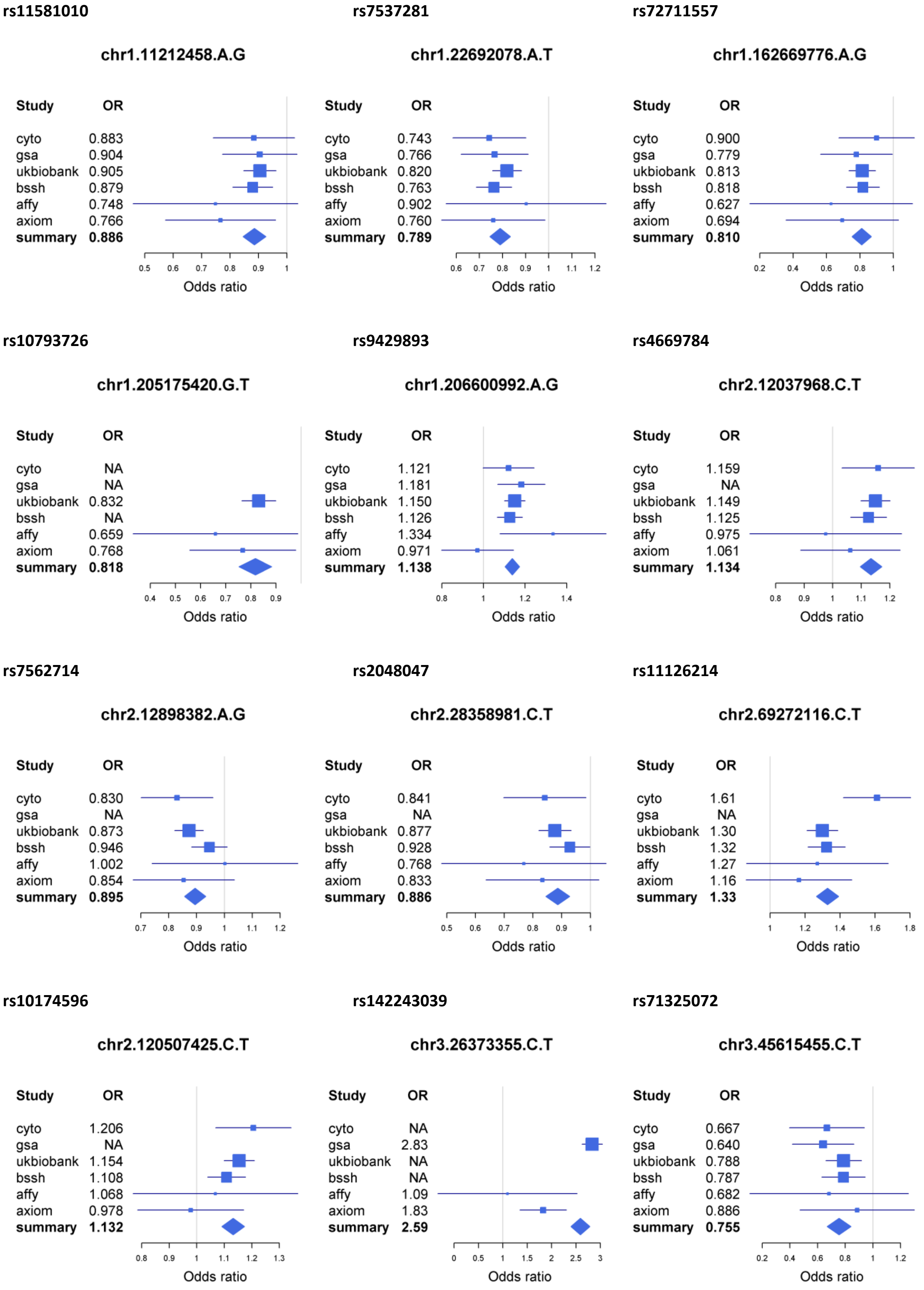


**Supplementary Figure 3.** Forest plots of the 85 significantly associated independent DD SNPs. SNPs are indicated with their chromosome and position. Effect sizes (OR) and 95% CI from the individual cohorts as well as from the meta-GWAS (summary) are shown. When a SNP was missing in a cohort, the effect size is indicated with “NA”.

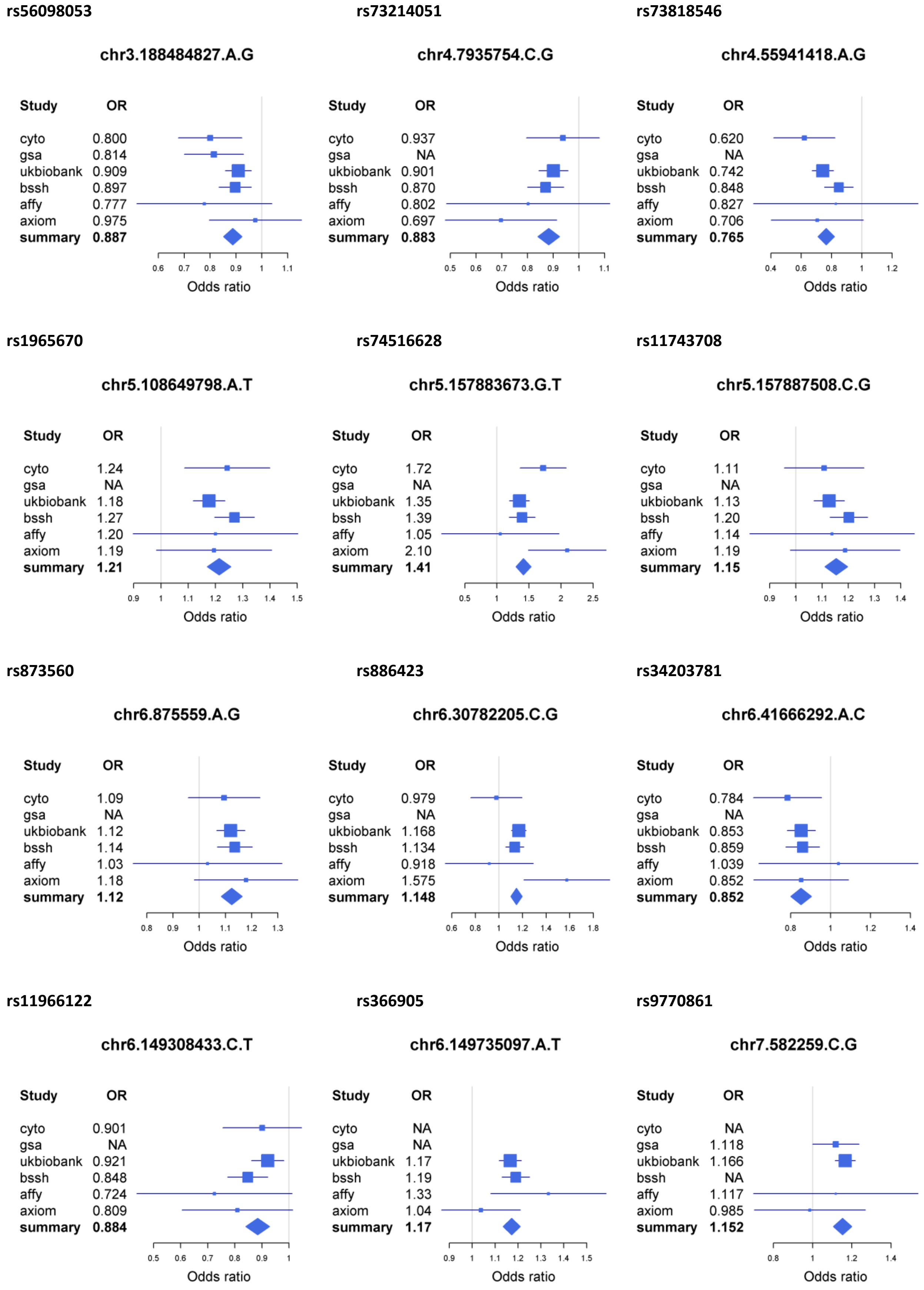


**Supplementary Figure 3** (continued)

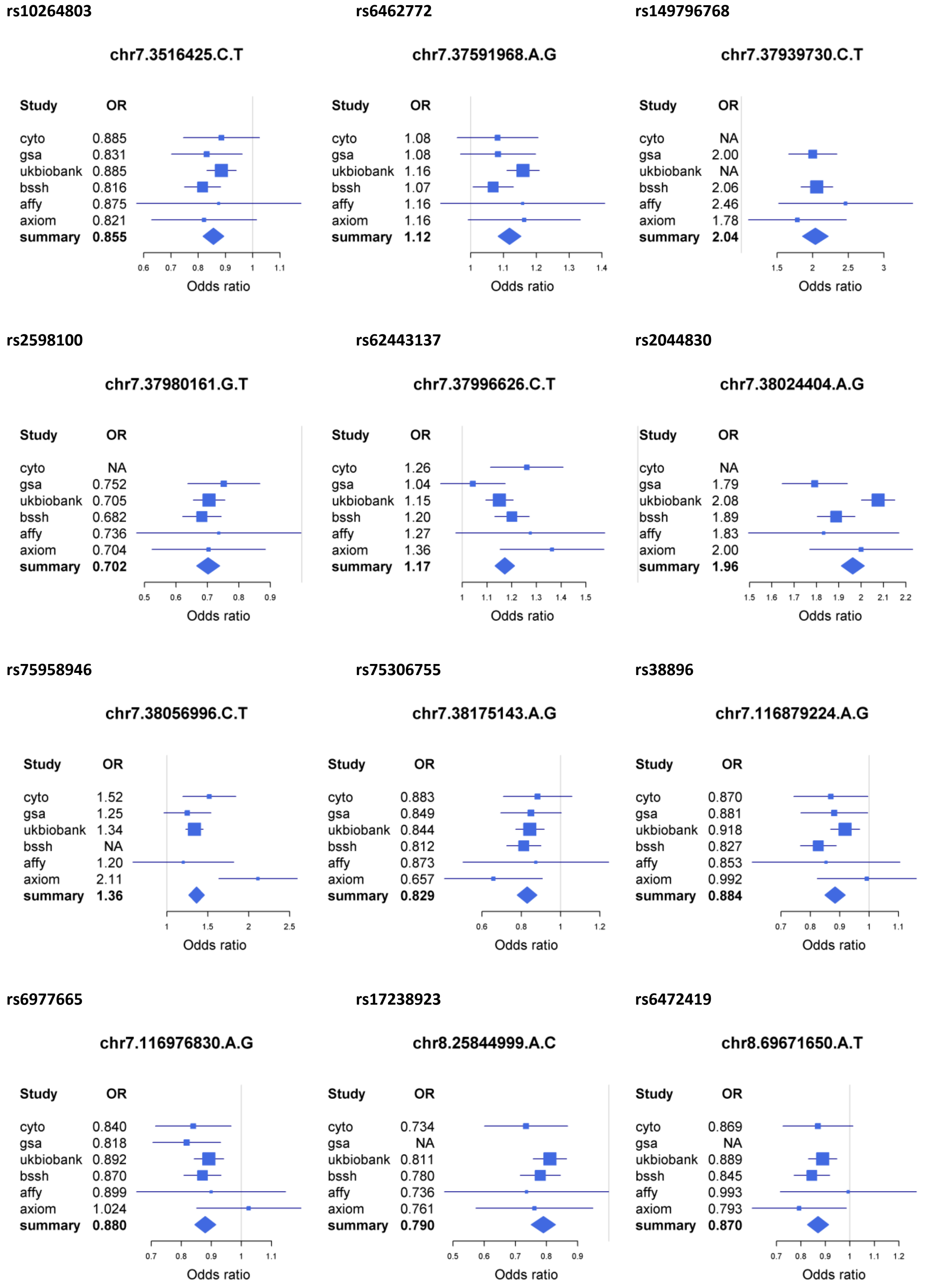


**Supplementary Figure 3** (continued)

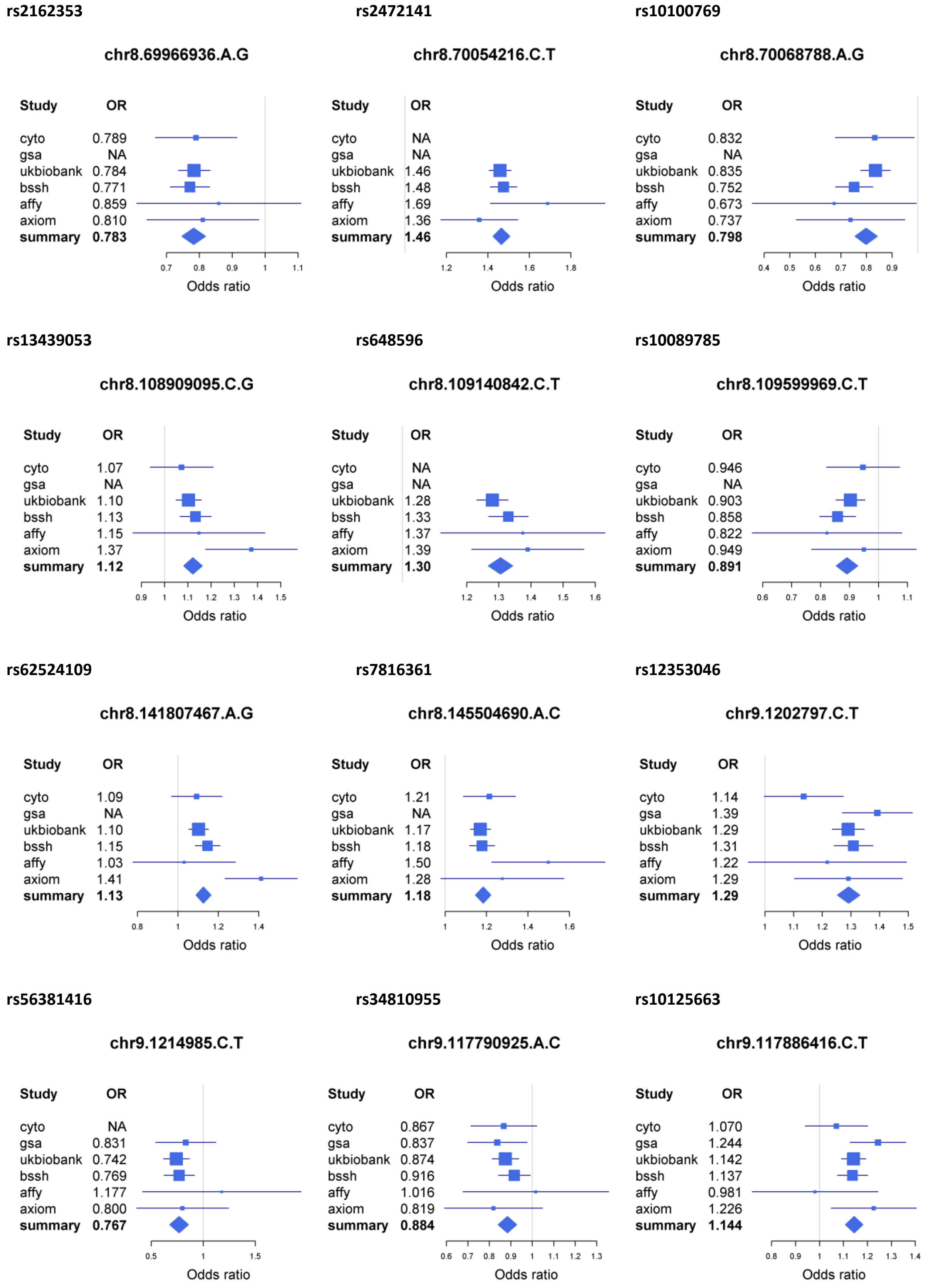


**Supplementary Figure 3** (continued)

rs1649199

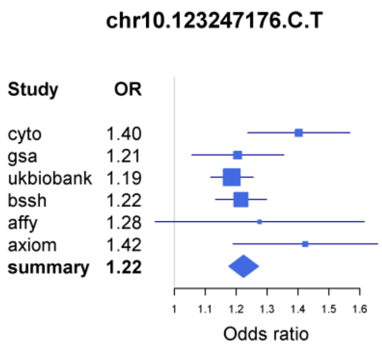


rs11200062

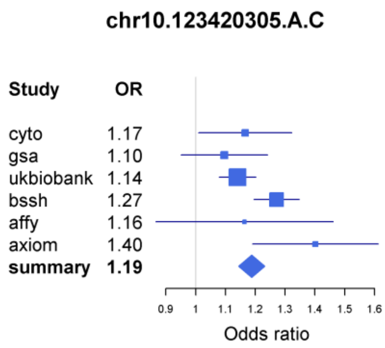


rs10831757

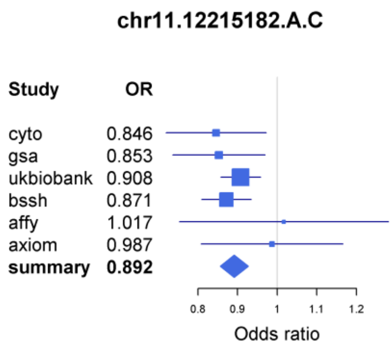


rs7307913

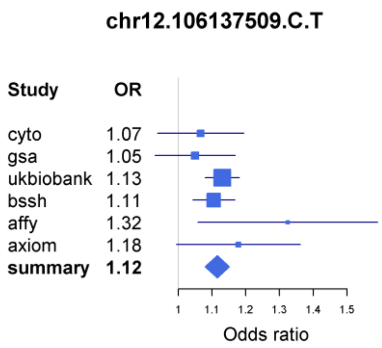


rs77660995

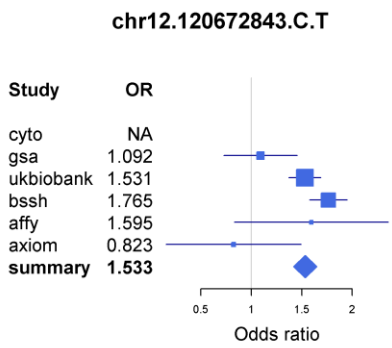


rs4942308

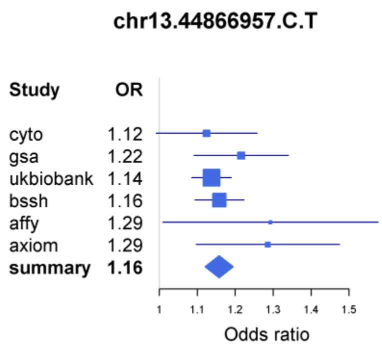


rs1042704

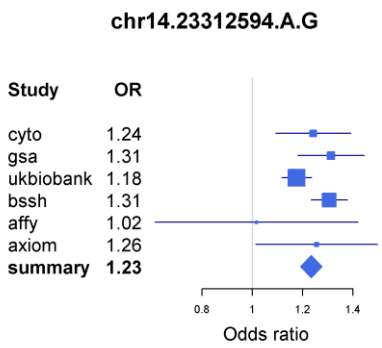


rs12881869

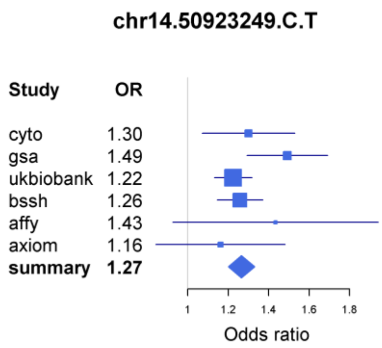


rs17791680

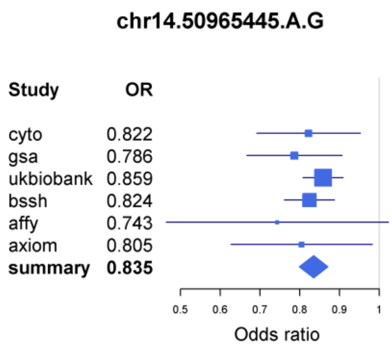


rs8032158

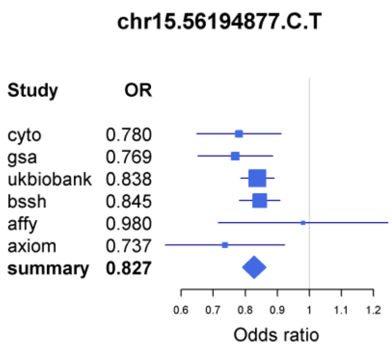


rs34412930

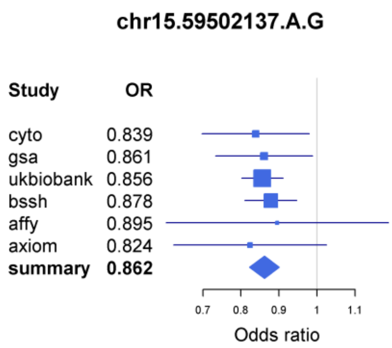


rs28522005

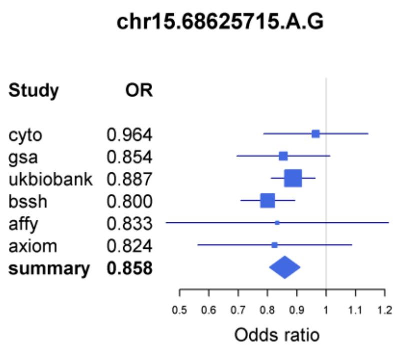


**Supplementary Figure 3** (continued)

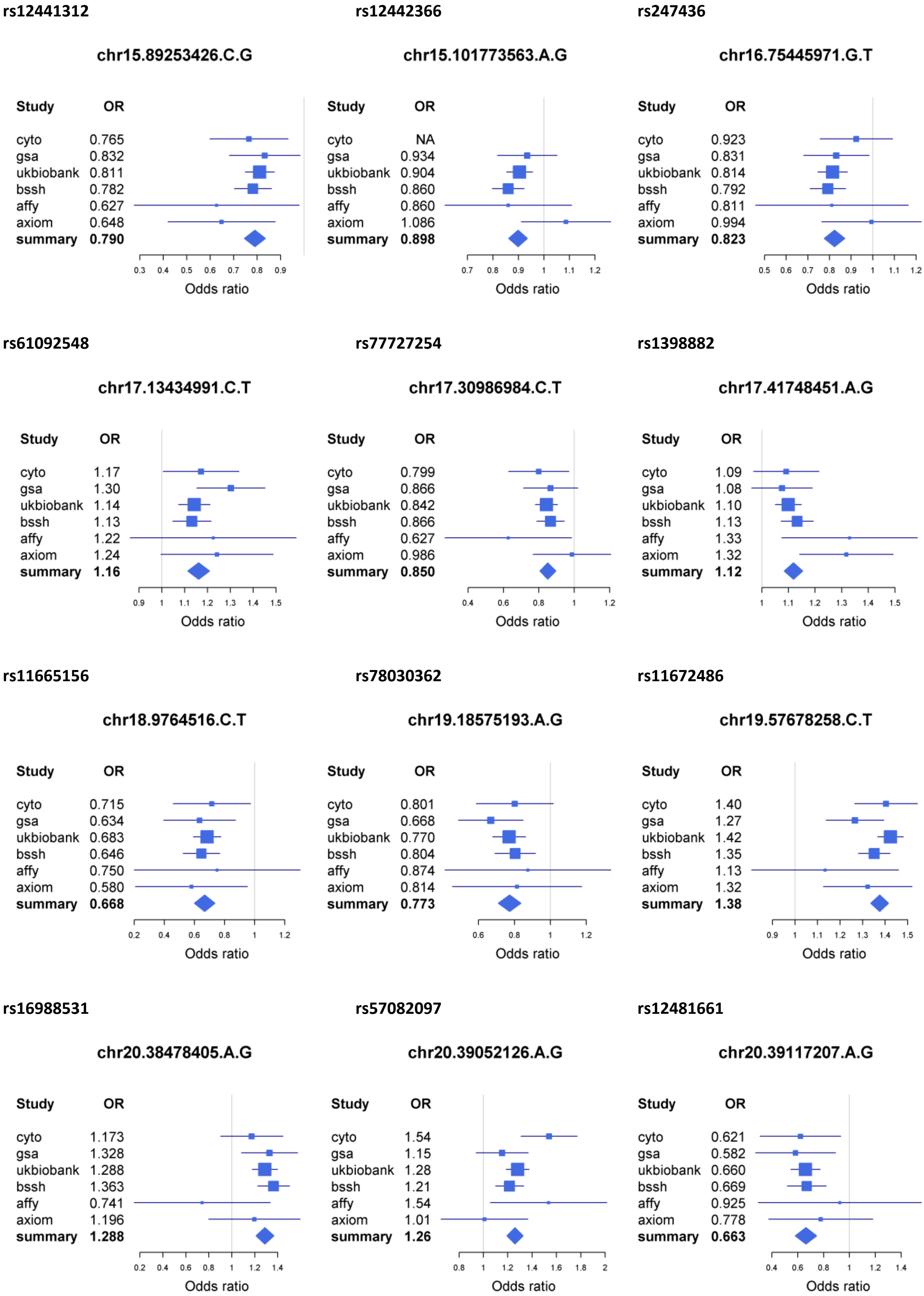


**Supplementary Figure 3** (continued)

rs181089987

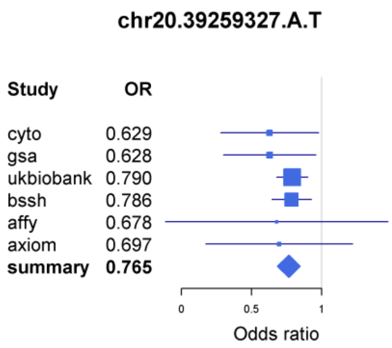


rs58716951

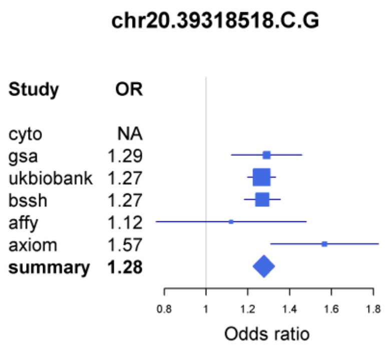


rs73130881

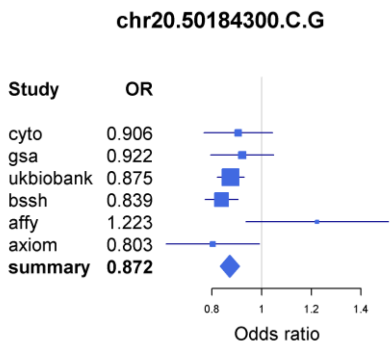


rs235931

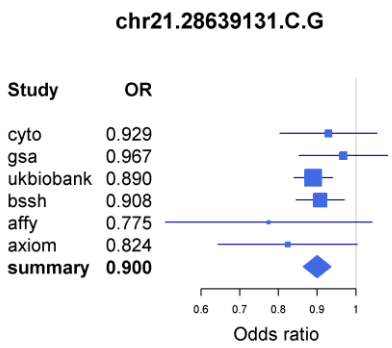


rs117999064

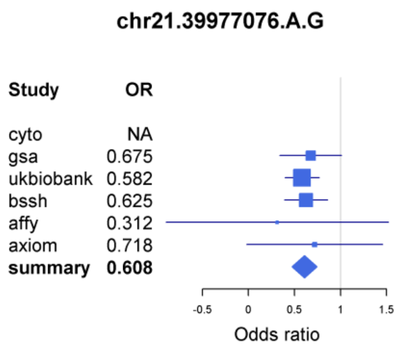


rs17573837

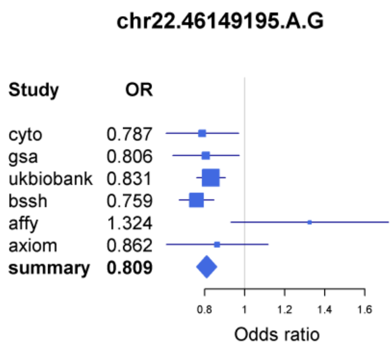


rs11704955

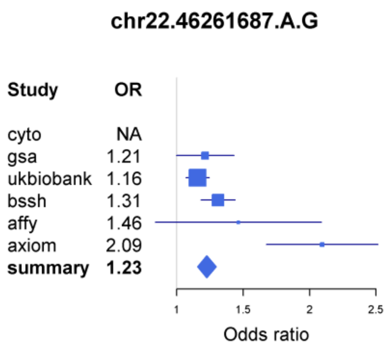


rs28363932

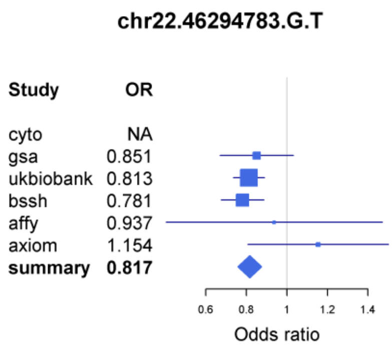


rs28755830

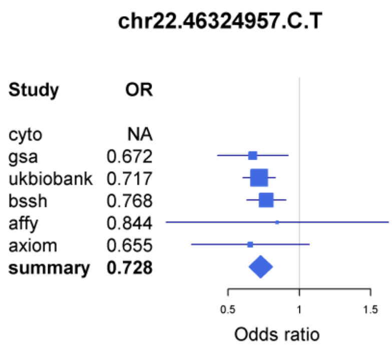


rs145039496

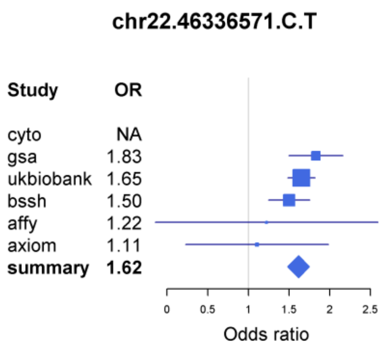


rs117707689

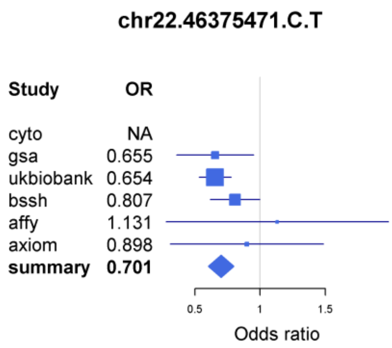


rs112116858

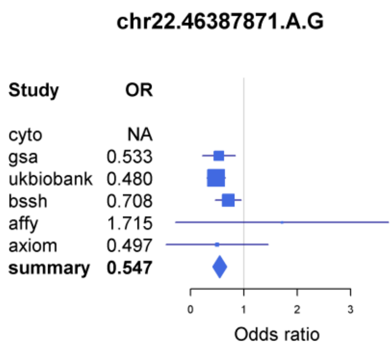


**Supplementary Figure 3** (continued)

rs9626908

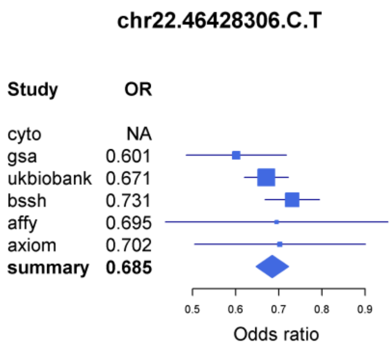


**Supplementary Figure 3** (continued)

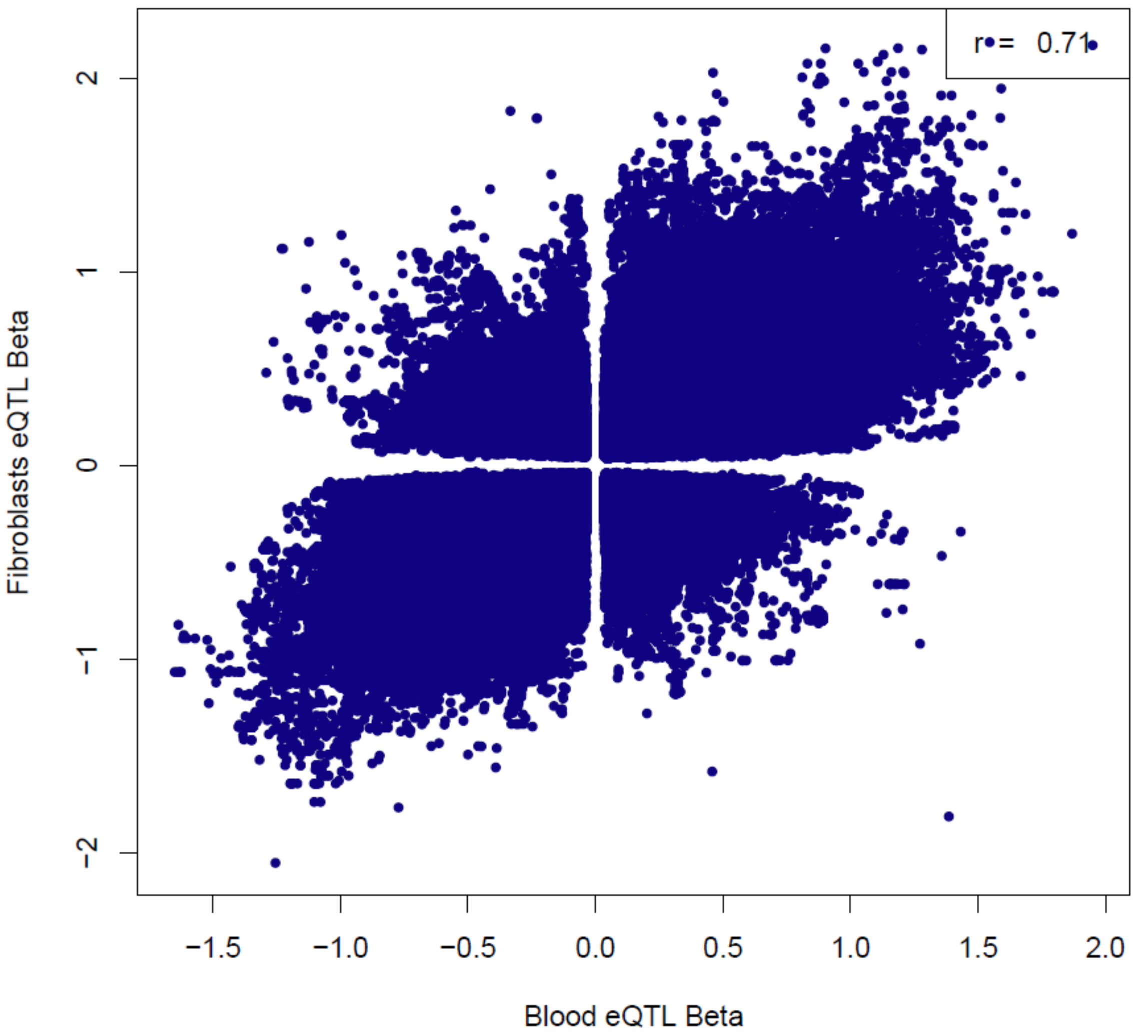


**Supplementary Figure 4.** Scatter plot of effect sizes for blood and fibroblasts FDR-controlled eQTLs (FDR < 0.05).

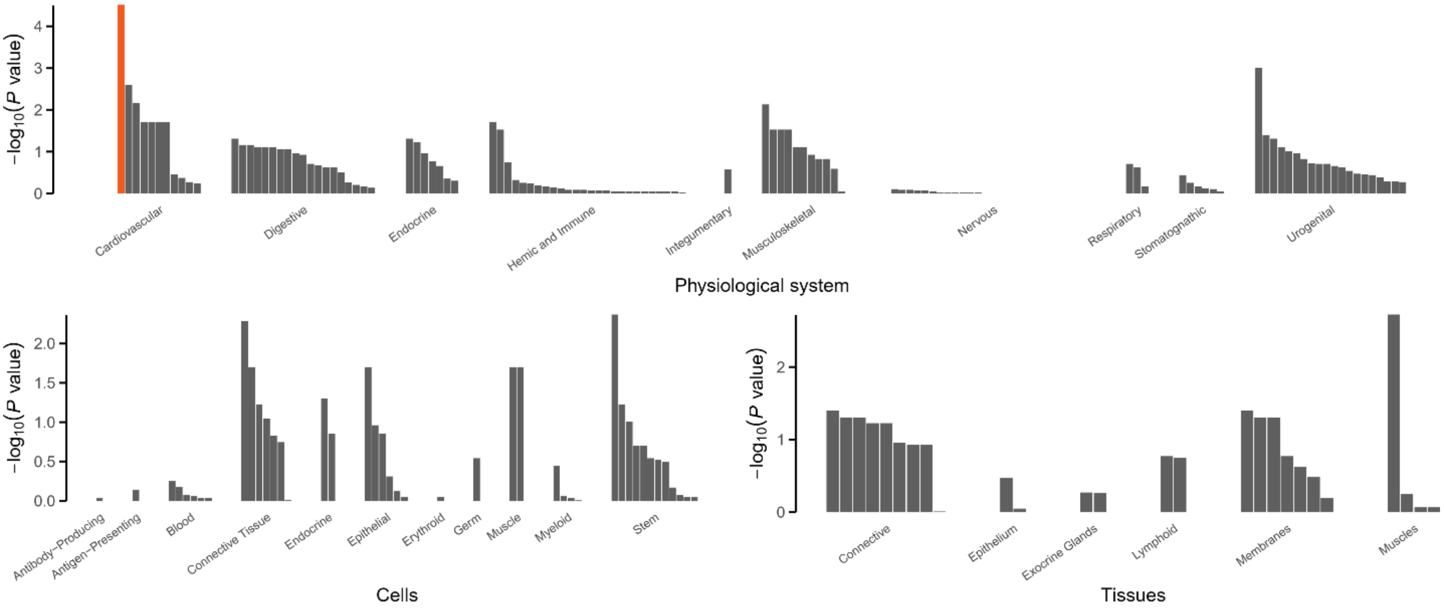


**Supplementary Figure 5.** DEPICT tissue enrichment results of 209 tissue/cell types. The colored bar is arteries with FDR < 0.01.

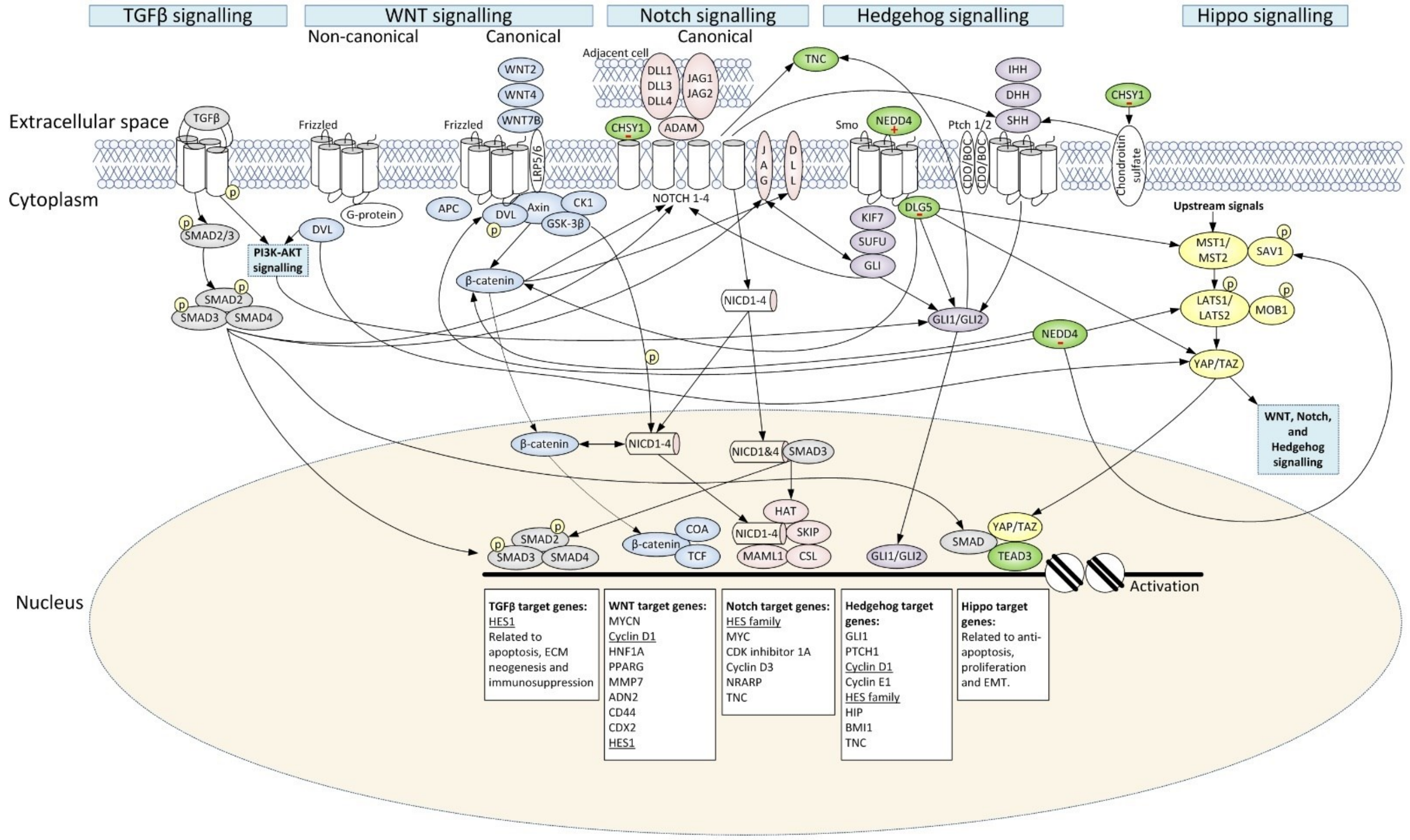


**Supplementary Figure 6.** A schematic representation of potential mechanisms of signalling pathways involved in Dupuytren's disease and the potential roles of prioritized genes *CHSY1*, *NEDD4*, *DLG5*, *TNC*, and *TEAD3* (green). Target genes shared between signalling pathways are underlined. Grey colored pathway: binding of TGFβ ligands to the TGFβ type II receptor catalyzes the phosporylation of the type I receptor. This complex activates intracellular effector proteins SMADs via phoshporylation. SMAD2 and SMAD3 bind to mediator SMAD4, causing this complex to translocate to the nucleus. Here, the SMAD transcriptional complex pairs with other transcription factors to regulate hundreds of genes. In fibroblasts the activation of TGFβ signaling results in repression of NOTCH3. Blue coloured pathway: WNT signalling constitutes of a non-canonical and canonical pathway. Non-canonical WNT signalling regulates inhibition of the canonical WNT signalling in addition to cytoskeletal rearrangement, and cell adhesion and movement. Activation of the WNT pathway by WNT ligands 2, 4 and 7B is mediated by phosphorylated Dishevelled (DVL) and results in accumulation of β-catenin due to disruption of a multiprotein destruction complex. After translocation to the nucleus, β-catenin Interacts with transcription factors to regulate WNT target genes including *Cyclin D1* and *Hes1*. β-catenin accumulation enables DVL to interact with transcriptional regulators, including YAP/TAZ, thereby influencing WNT, NOTCH, and Hedgehog signalling. NEDD4 attaches ubiquitins to β-catenin and DVL, usually inducing their proteasomal degradation. DLG5 can bind to β-catenin, however with unknown consequences. Red coloured pathway: CHSY1 negatively regulates Notch signalling via interaction with NOTCH receptors. Other NOTCH receptor ligands, JAG1, JAG2, DLL1, DLL3, and DLL4, are mostly transmembrane proteins of adjacent cells. Binding of these ligands to receptors NOTCH1, NOTCH2, NOTCH3 and/or NOTCH4 causes cleavage of the NOTCH receptor, mediated by ADAM proteins. This results in the release of NOTCH intracellular domain (NICD), which interacts with transcriptional regulators to stimulate gene expression. NICD interacts with β-catenin and SMAD proteins: NICD4/SMAD3 interaction has a negative impact on TGFβ-dependent transcription, while NICD-1/SMAD3 interaction enhances both TGFβ-dependent and NOTCH-dependent gene transcription. Moreover, glycogen synthase kinase 3β (GSK-3β) is able to phosphorylate NICD1 and NICD2. Activation of TGFβ signaling results in upregulation JAG1, which in turn activates the Notch pathway. TGFβ signaling also results in repression of NOTCH3. The WNT pathway may regulate the expression of the NOTCH ligand DLL1. Direct interaction between β-catenin and NOTCH1 reduces NOTCH1 ubiquitination, resulting in increased target gene expression. The extracellular glycoprotein TNC is also a target of NOTCH signalling and upregulates FAK/MAPK signalling. Upregulation of Hedgehog (Hh) transcription factors GLI1 results in upregulation of both JAG1 and NOTCH1 receptor mRNAs. JAG1 and GLI2 positively regulate each other's mRNA levels. Moreover, activation of NOTCH signalling induces the expression of Sonic Hh (SHH). Purple coloured pathway: binding of Hh ligands (SHH, Indian Hh [IHH], or Desert Hh [DHH]) to receptor patched (Ptch1/2) depresses its inhibitory activity against Smoothened (SMO). This leads to activation and nuclear localization of GLI transcription factors driving target gene expression. Binding of NEDD4 to SMO positively regulates Hh signalling. Cytoplasmic DLG5 is a binding partner of activated SMO upon Hh pathway activation. PI3K/Akt signalling can activate GLI signalling. TNC is a target gene of GLI1 and GLI2 and promotes fibroblast proliferation through Integrin/Focal Adhesion Kinase/ MAP kinase signalling. The extracellular enzyme CHSY1 promotes Hh signalling by increasing cell surface chondroitin sulfate, which promotes SHH binding and signalling. Yellow coloured pathway: Hippo pathway core kinase complexes are MST1/2 and LATS1/2, binding with SAV1 and MOB1, respectively. These kinases regulate downstream transcriptional co-activators YAP1 and TAZ. When these core kinase complexes become inactive, YAP and TAZ translocate to the nucleus where they associate with transcription factors such as TEAD. DLG5 plays a critical role in the formation of kinase complexes and interacts genetically with YAP1/TAZ. NEDD4 negatively regulates Hippo signalling through destabilisation of LATS and SAV1, inhibiting Hippo signalling. Hippo signalling crosstalks with WNT, NOTCH, and Hedgehog signalling through mechanisms yet unknown.

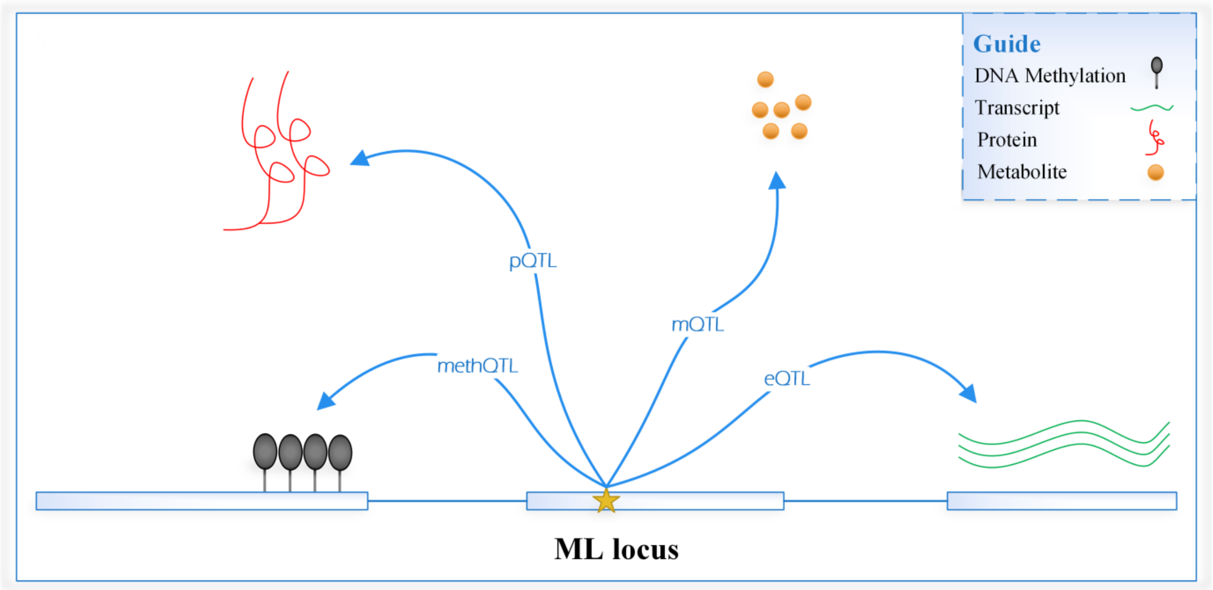


**Supplementary Figure 7.** Schematic illustration of loci with multiple molecular associations (ML). MethQTL, eQTL, pQTL, and mQTL indicate methylation, expression, protein, and metabolite Quantitative Trait Loci, respectively.

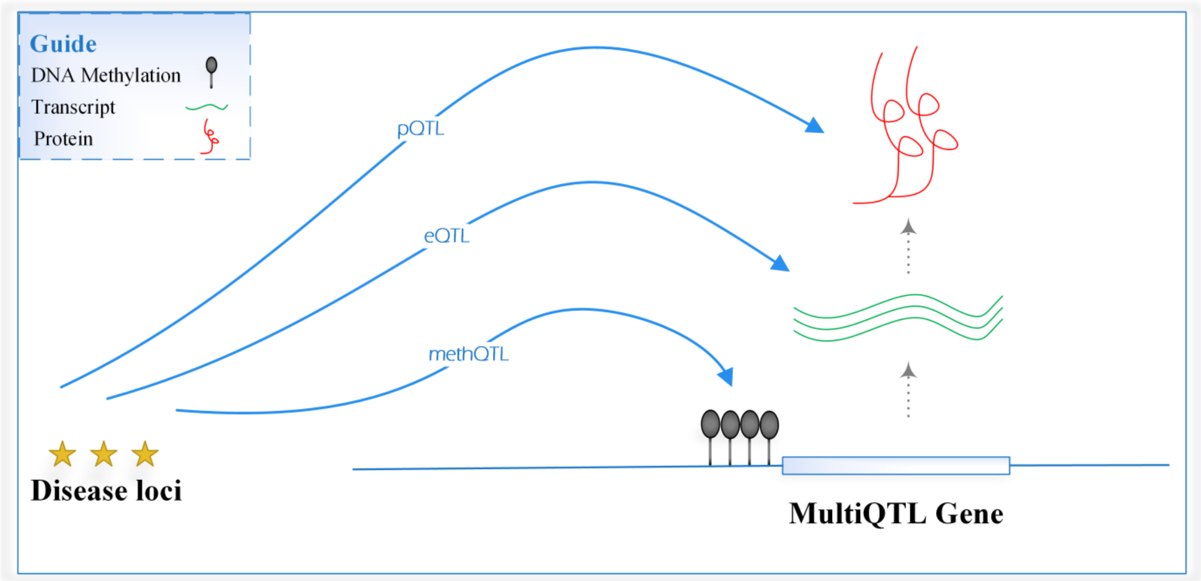


**Supplementary Figure 8.** Schematic illustration of downstream genes associated with disease loci by evidence from multiple molecular layers (MultiQTL). MethQTL, eQTL, and pQTL indicate methylation, expression, and protein Quantitative Trait Loci, respectively.

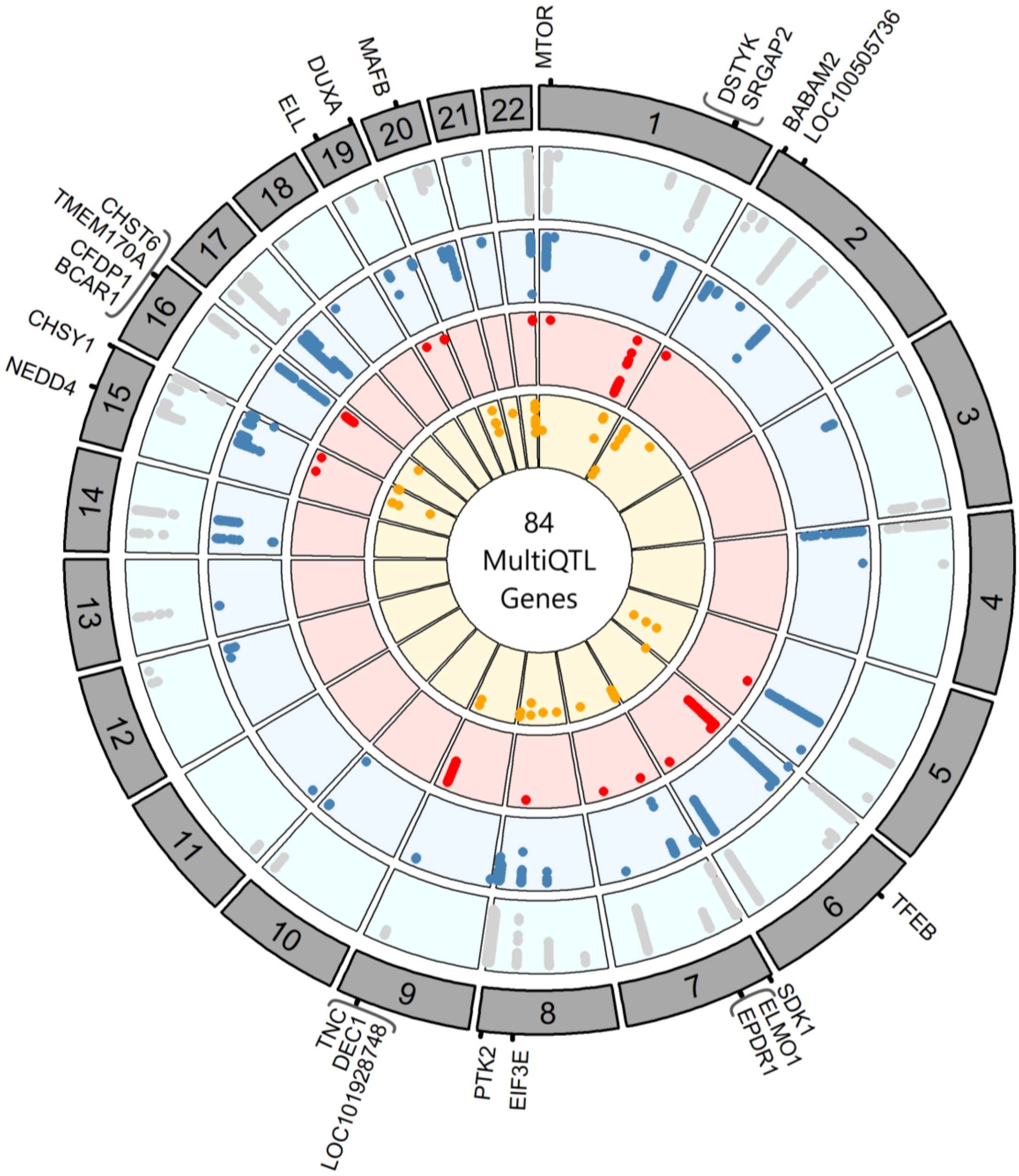


**Supplementary Figure 9**. Circular visualization of all multi-layer molecular associations across DD genomic loci ($r^2 > 0.5$). Only highly correlated ($r^2 > 0.8$) loci with multi-layer molecular associations are annotated. Outer to inner layers represent: genome (dark grey), epigenome (azure), transcriptome (light blue), proteome (light red), and metabolome (light orange). Dots represent p-value of associations for disease loci with molecular quantitative traits of DNA methylation, gene expression, protein, and metabolite levels on logit scale. Only p-values < $1x10^{-5}$ are shown.

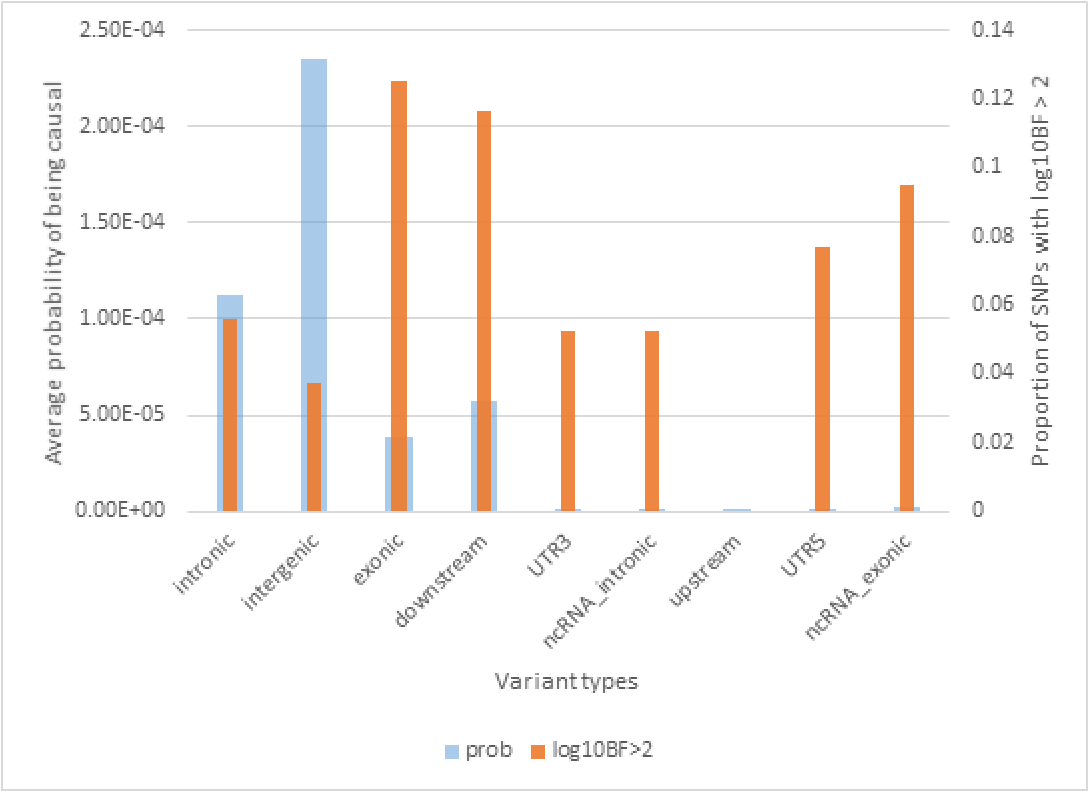


**Supplementary Figure 10.** FINEMAP analysis showing the average probability of being causal (left y-axis) and the proportion of SNPs with considerable evidence of being causal (right y-axis) plotted for different types of variants.

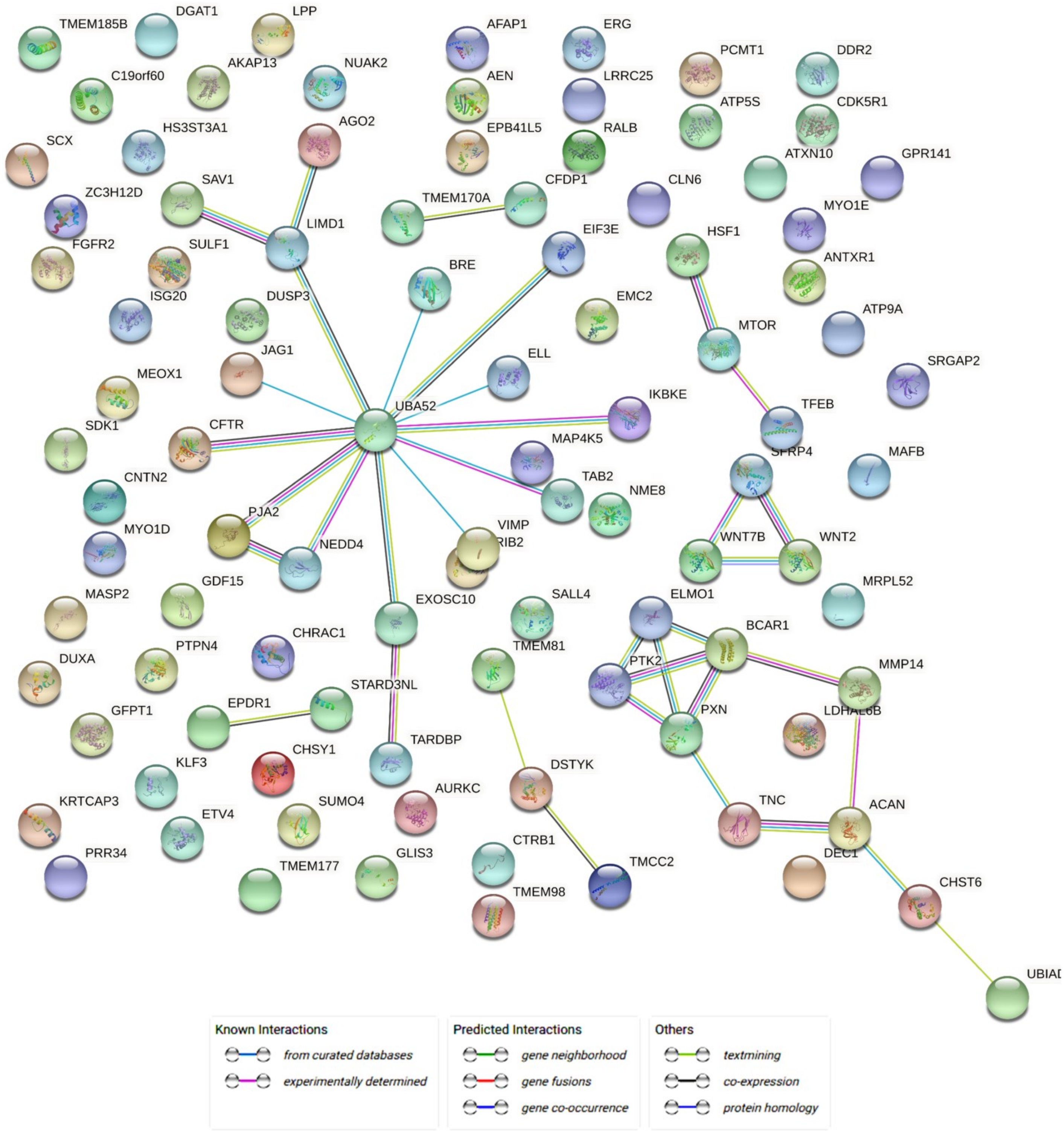


**Supplementary Figure 11**. STRING protein-protein interaction network of the 119 prioritized genes with a confidence cut-off of ≥ 0.7.

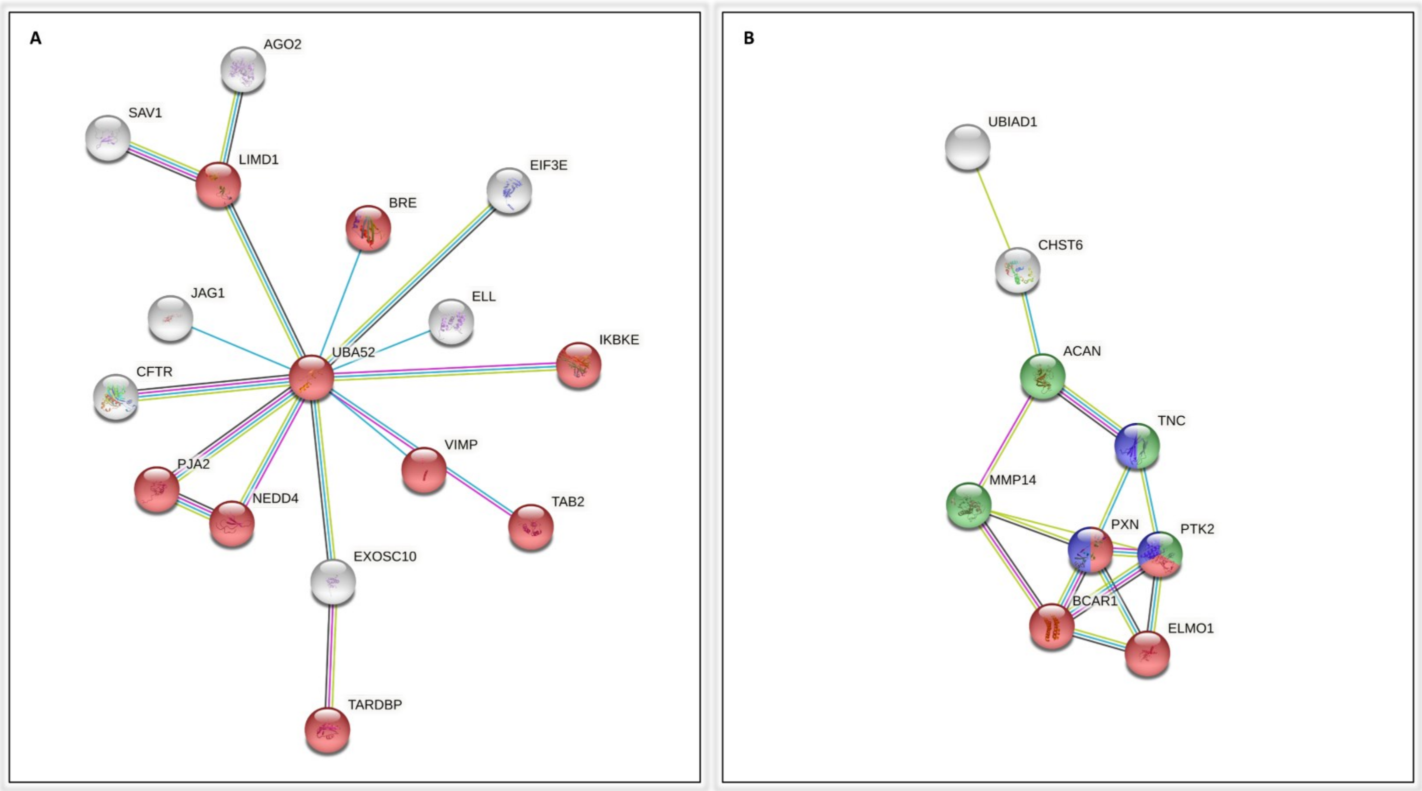


**Supplementary Figure 12**. Major connected components of the STRING protein-protein interaction network of the 119 prioritized genes. Colored nodes are those involved in major significant pathways of the corresponding subnetwork. Red in figure A indicates nodes involved in response to stress. In figure B, blue is for the extracellular matrix organization, red for the bacterial invasion of the epithelial cells, and green is for the human papillomavirus infection.

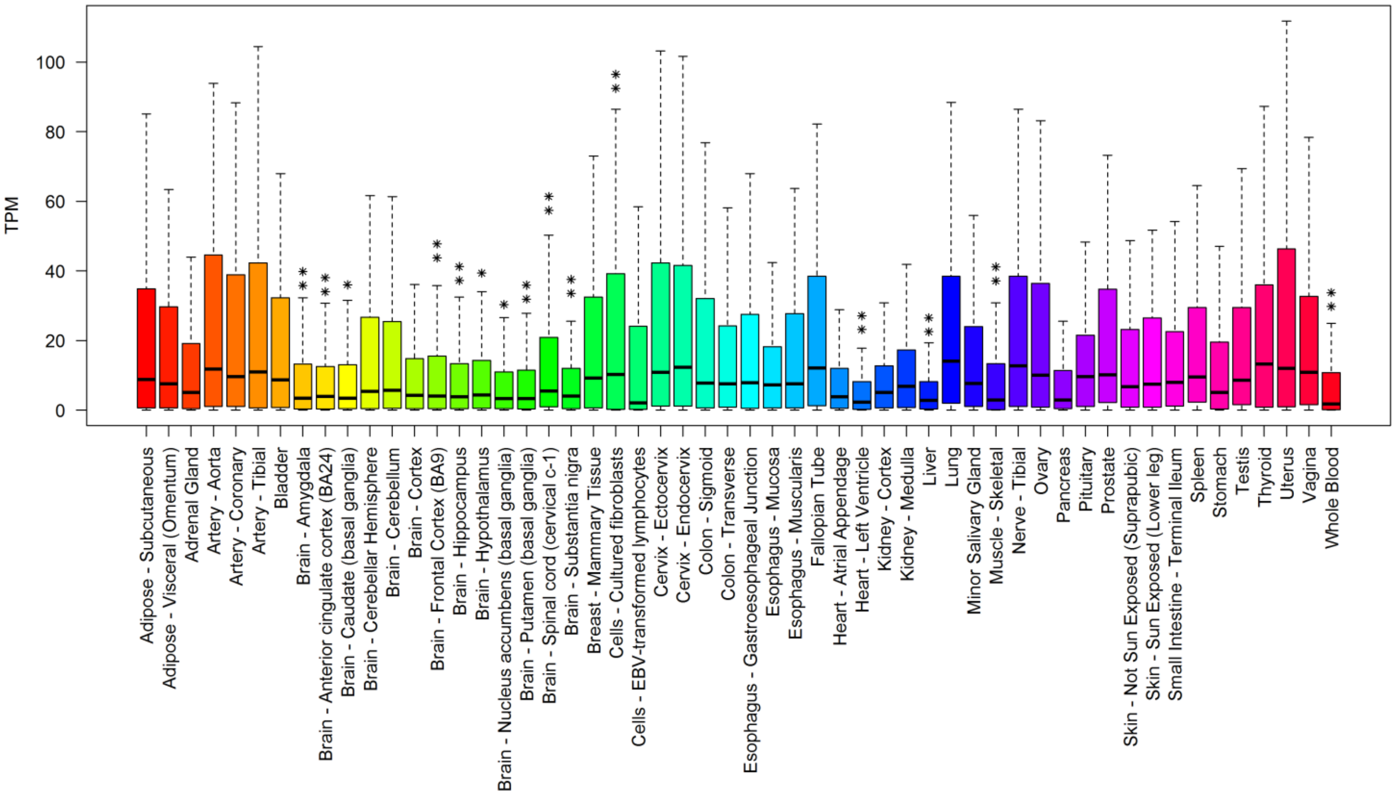


**Supplementary Figure 13.** Distribution of gene expression values of 119 prioritized genes for DD, and tissue enrichment results across 54 human tissues from GTEx v8 database. A single star indicates FDR < 0.05 and a double star indicates FDR < 0.01.

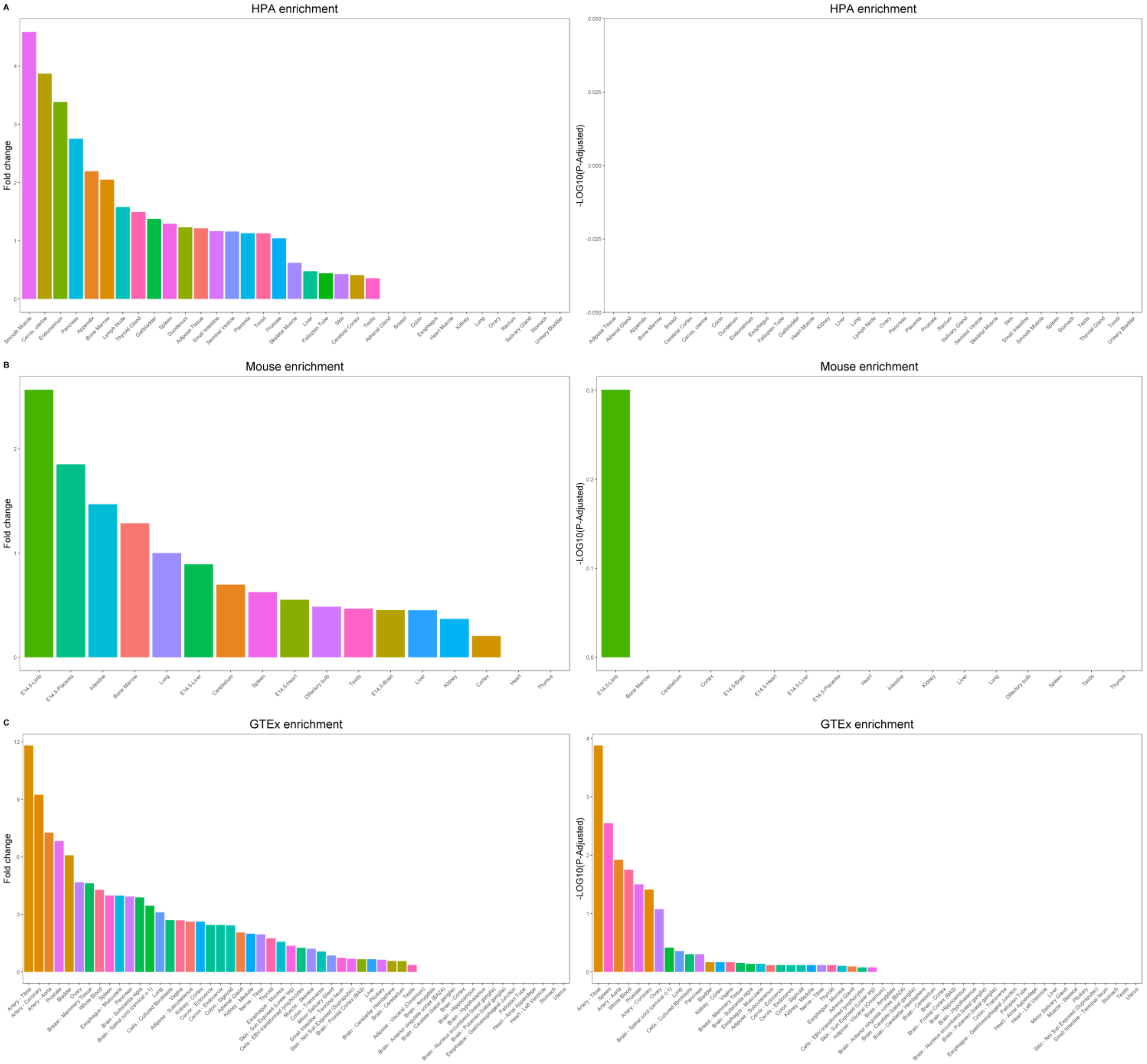


**Supplementary Figure 14.** Enrichment of tissue-specific genes in our 119 prioritized genes based on different datasets. HPA: Human Protein Atlas; GTEx: Genotype-Tissue Expression database.

## REFERENCES


1. Shah TS, Liu JZ, Floyd JAB, Morris JA, Wirth N, Barrett JC, et al. optiCall: a robust genotype-calling algorithm for rare, low-frequency and common variants. Bioinformatics. 2012 Jun;28(12):1598–603.

2. Purcell S, Neale B, Todd-Brown K, Thomas L, Ferreira MAR, Bender D, et al. PLINK: A tool set for whole-genome association and population-based linkage analyses. Am J Hum Genet. 2007;81(3):559–75.

3. Auton A, Abecasis GR, Altshuler DM, Durbin RM, Bentley DR, Chakravarti A, et al. A global reference for human genetic variation. Nature. 2015;526(7571):68–74.

4. Yang J, Lee SH, Goddard ME, Visscher PM. GCTA: a tool for genome-wide complex trait analysis. Am J Hum Genet. 2011 Jan;88(1):76–82.

5. R Core Team. R: A language and environment for statistical computing. 2014;

6. Das S, Forer L, Schönherr S, Sidore C, Locke AE, Kwong A, et al. Next-generation genotype imputation service and methods Sayantan. Nat Genet. 2016;48(10):1284–7.

7. McCarthy S, Das S, Kretzschmar W, Delaneau O, Wood AR, Teumer A, et al. A reference panel of 64,976 haplotypes for genotype imputation. Nat Genet. 2016;48(10):1279–83.

8. Danecek P, Bonfield JK, Liddle J, Marshall J, Ohan V, Pollard MO, et al. Twelve years of SAMtools and BCFtools. Gigascience. 2021 Feb;10(2).

9. Choi SW, O'Reilly PF. PRSice-2: Polygenic Risk Score software for biobank-scale data. Gigascience. 2019 Jul;8(7):giz082.

10. Pers TH, Karjalainen JM, Chan Y, Westra HJ, Wood AR, Yang J, et al. Biological interpretation of genome-wide association studies using predicted gene functions. Nat Commun. 2015 Jan;6:5890.

11. Zhu Z, Zhang F, Hu H, Bakshi A, Robinson MR, Powell JE, et al. Integration of summary data from GWAS and eQTL studies predicts complex trait gene targets. Nat Genet. 2016;48(5):481–7.

12. Võsa U, Claringbould A, Westra HJ, Bonder MJ, Deelen P, Zeng B, et al. Unraveling the polygenic architecture of complex traits using blood eQTL meta-analysis. bioRxiv. 2018;1–57.

13. The GTEx Consortium atlas of genetic regulatory effects across human tissues. Science. 2020 Sep;369(6509):1318–30.

14. Staley JR, Blackshaw J, Kamat MA, Ellis S, Surendran P, Sun BB, et al. PhenoScanner: A database of human genotype-phenotype associations. Bioinformatics. 2016;32(20):3207–9.

15. Benner C, Spencer CCA, Havulinna AS, Salomaa V, Ripatti S, Pirinen M. FINEMAP: Efficient variable selection using summary data from genome-wide association studies. Bioinformatics. 2016;32(10):1493–501.

16. Chang CC, Chow CC, Tellier LCAM, Vattikuti S, Purcell SM, Lee JJ. Second-generation PLINK: Rising to the challenge of larger and richer datasets. Gigascience. 2015;4(1):1–16.

17. Montojo J, Zuberi K, Rodriguez H, Kazi F, Wright G, Donaldson SL, et al. GeneMANIA Cytoscape plugin: fast gene function predictions on the desktop. Bioinformatics. 2010;26(22):2927–8.

18. Szklarczyk D, Gable AL, Lyon D, Junge A, Wyder S, Huerta-Cepas J, et al. STRING v11: protein-protein association networks with increased coverage, supporting functional discovery in genome-wide experimental datasets. Nucleic Acids Res. 2019 Jan;47(D1):D607–13.

19. Jain A, Tuteja G. TissueEnrich: Tissue-specific gene enrichment analysis. Bioinformatics. 2019 Jun;35(11):1966–7.

20. Shen Y, Yue F, McCleary DF, Ye Z, Edsall L, Kuan S, et al. A map of the cis-regulatory sequences in the mouse genome. Nature. 2012 Aug;488(7409):116–20.
