# Supplementary material for: A genome-wide association meta-analysis implicates Hedgehog and Notch signaling in Dupuytren’s disease
Source: Nat Commun. 2024 Jan 3;15:199. doi: 10.1038/s41467-023-44451-0 (PMC10764787; doi:10.1038/s41467-023-44451-0)
Supplement: Supplementary file 2 — Description of Additional Supplementary Files [file 41467_2023_44451_MOESM2_ESM.docx]

**Supplementary Data Legends**

File Name: Supplementary Data 1
Description: Supplementary Data 1. Description of study cohorts.

File Name: Supplementary Data 2
Description: Supplementary Data 2. Quality control output parameters of GWAS summary statistics per cohort and the meta-GWAS.

File Name: Supplementary Data 3
Description: Supplementary Data 3. Independent identified loci genome-wide significantly (p<5x10^-8) associated with DD in the meta-analysis of GWASs, using METALs fixed effects inverse variance weighting method. The novel loci and/or variants are highlighted in bold.

File Name: Supplementary Data 4
Description: Supplementary Data 4. Meta-GWAS results, acquired using METALs fixed effects inverse variance weighting method, from this study for the 27 previously identified variants in the FinnGen cohort. (10, https://r5.finngen.fi/pheno/M13_DUPUTRYEN)

File Name: Supplementary Data 5
Description: Supplementary Data 5. Replication and meta-analysis (using METALs fixed effects inverse variance weighting method) of our meta-GWAS genome-wide significant loci in FinnGen GWAS summary statistics. SNPs with a one-sided p-value <0.000588 (=0.05/85, i.e. Bonferroni correction) were considered replicated.

File Name: Supplementary Data 6
Description: Supplementary Data 6. The results of in silico sequencing of 85 SNPs for DD, using correlation testing between SNPs for linkage disequilibrium (r2>0.5) and ANNOVAR for functional annotation. 'AF alt all EUR' indicates the allele frequency of the alternative allele (A2) in the European population.

File Name: Supplementary Data 7
Description: Supplementary Data 7. Gene expression association of the 119 prioritized genes with Dupuytren's disease in fibroblasts, using a summary-data-based Mendelian Randomization analysis with a Bonferroni corrected significance level of <3.23x10^-6 (i.e. 0.05/15,491), and a heterogeneity in dependent instruments test with a Bonferroni corrected significance of ≥2.08x10^-3 (i.e., 0.05/number of SMR significant genes).

File Name: Supplementary Data 8
Description: Supplementary Data 8. Results from the FINEMAP analysis (analysing the 55 genomic regions via shotgun stochastic search algorithms) listing the most likely causal SNPs for loci identified for Dupuytren disease.

File Name: Supplementary Data 9
Description: Supplementary Data 9. Data-driven Expression Prioritized Integration for Complex Traits (DEPICT) significant (FDR < 0.05) gene set enrichment results using Dupuytren GWAS summary statistics. A p-value threshold of 5x10^-8, an r^2 of 0.1 (as an LD metric), and a physical distance of 500 kb for clumping were used.

File Name: Supplementary Data 10
Description: Supplementary Data 10. Functional enrichment results of the 119 prioritized genes for Dupuytren's disease using GeneMANIA

File Name: Supplementary Data 11
Description: Supplementary Data 11. False Discovery Rates (FDR) of the number of DD genes expressed in 54 GTEx tissues, based on 10,000 permutation runs.

File Name: Supplementary Data 12
Description: Supplementary Data 12. Significant Summary-data-based Mendelian Randomization (SMR) analysis results of Dupuytren GWAS using the GTEx dataset of fibroblast cis-eQTLs in European individuals. We performed a heterogeneity in dependent instruments (HEIDI) test to filter out confouned results and used Bonferroni corrected significance levels of <3.23x10-6 (i.e. 0.05/15,491) for SMR ≥2.08x10-3 (i.e., 0.05/number of SMR significant genes) for the HEIDI test.

File Name: Supplementary Data 13
Description: Supplementary Data 13. Significant Summary-data-based Mendelian Randomization (SMR) analysis results of Dupuytren GWAS using the eQTLGen dataset of whole blood cis-eQTLs. We performed a heterogeneity in dependent instruments (HEIDI) test to filter out confouned results and used Bonferroni corrected significance levels of <3.23x10-6 (i.e. 0.05/15,491) for SMR ≥2.08x10-3 (i.e., 0.05/number of SMR significant genes) for the HEIDI test.

File Name: Supplementary Data 14
Description: Supplementary Data 14. Cell-population specific gene analysis using SNPsea. For each SNP and cell type, the gene with greatest specificity to that cell type and its specificity score are given, adjusted for the number of genes overlapping the given SNP’s linkage interval. SNPs with shared genes (column linked SNPs) were merged into a single locus (represented by column SNP) to avoid multiple counting of genes. A lower specificity score means that the gene is more specific for that cell type.

File Name: Supplementary Data 15
Description: Supplementary Data 15. Genetic correlation between DD and resembling or associated traits and disorders, using LD score regression and a Bonferroni‐corrected threshold of α=0.05/14=0.0036.

File Name: Supplementary Data 16
Description: Supplementary Data 16. A list of genes discovered in at least 3 gene prioritization analyses and replicated in SMR fibroblast analysis, and genes identified by SMR fibroblast analysis.

File Name: Supplementary Data 17
Description: Supplementary Table 17. List of prioritized genes from all bioinformatic follow-up analyses.

File Name: Supplementary Data 18
Description: Supplementary Data 18. Co-regulation analysis results acquired using using DEPICT and its accompanying expression dataset of 77,840 samples. Reported are DEPICT significant (false discovery rate ≤ 0.01) gene prioritization results using Dupuytren GWAS summary statistics. We used a p-value threshold of 5x10-8, an r^2 of 0.1 (as an LD metric), and a physical distance of 500 kb for clumping.

File Name: Supplementary Data 19
Description: Supplementary Data 19. Genes with variants showing associations with three or four molecular layers.

File Name: Supplementary Data 20
Description: Supplementary Data 20. Genes having more than one source of association with variants linked to (r2>0.8) genomic loci for Dupuytren's disease (multiQTL).

File Name: Supplementary Data 21
Description: Supplementary Data 21. Functional enrichment results of the 73 prioritized genes within Dupuytren's GWAS loci (r2>0.5) using GeneMANIA

File Name: Supplementary Data 22
Description: Supplementary Data 22. Functional enrichment results of the 23 prioritized genes for Dupuytren's disease, with more than one source of evidence, using GeneMANIA

File Name: Supplementary Data 23
Description: Supplementary Data 23. Functional enrichment results of the 119 prioritized genes for Dupuytren's disease using STRING

File Name: Supplementary Data 24
Description: Supplementary Data 24. Functional enrichment results of the first and second connected components of the prioritized genes for Dupuytren's disease using STRING
