# Supplementary material for: A genome-wide association meta-analysis implicates Hedgehog and Notch signaling in Dupuytren’s disease
Source: Nat Commun. 2024 Jan 3;15:199. doi: 10.1038/s41467-023-44451-0 (PMC10764787; doi:10.1038/s41467-023-44451-0)
Supplement: Supplementary file 27 — Reporting Summary [file 41467_2023_44451_MOESM27_ESM.pdf]

nature portfolio

Corresponding author(s): S. A. Riesmeijer

Last updated by author(s): 11/25/2023

Reporting Summary

Nature Portfolio wishes to improve the reproducibility of the work that we publish. This form provides structure for consistency and transparency in reporting. For further information on Nature Portfolio policies, see our Editorial Policies and the Editorial Policy Checklist.

Statistics

For all statistical analyses, confirm that the following items are present in the figure legend, table legend, main text, or Methods section.

n/a	Confirmed	
☐	☒	The exact sample size (*n*) for each experimental group/condition, given as a discrete number and unit of measurement
☐	☒	A statement on whether measurements were taken from distinct samples or whether the same sample was measured repeatedly
☐	☒	The statistical test(s) used AND whether they are one- or two-sided<br>*Only common tests should be described solely by name; describe more complex techniques in the Methods section.*
☐	☒	A description of all covariates tested
☐	☒	A description of any assumptions or corrections, such as tests of normality and adjustment for multiple comparisons
☐	☒	A full description of the statistical parameters including central tendency (e.g. means) or other basic estimates (e.g. regression coefficient) AND variation (e.g. standard deviation) or associated estimates of uncertainty (e.g. confidence intervals)
☐	☒	For null hypothesis testing, the test statistic (e.g. *F*, *t*, *r*) with confidence intervals, effect sizes, degrees of freedom and *P* value noted<br>*Give P values as exact values whenever suitable.*
☒	☐	For Bayesian analysis, information on the choice of priors and Markov chain Monte Carlo settings
☒	☐	For hierarchical and complex designs, identification of the appropriate level for tests and full reporting of outcomes
☐	☒	Estimates of effect sizes (e.g. Cohen's *d*, Pearson's *r*), indicating how they were calculated

Our web collection on statistics for biologists contains articles on many of the points above.

Software and code

Policy information about availability of computer code

Data collection	We used data from six already existing cohorts comprised of DD cases and healthy controls from the Netherlands, United Kingdom (UK), and Germany. The cases were individuals of European ancestry who had been diagnosed with DD by a plastic or hand surgeon and/or who had undergone surgical treatment for DD. Controls were population-based subjects from the Lifelines cohort study (the Netherlands), from the UK Household Longitudinal Study (UK), the UK Biobank initiative (UK), and from the PopGen and KORA studies (Germany) with no known diagnosis of DD.

Data analysis

The software used for data analysis is freely available and mostly we used standard setting. The quality control (QC) pipeline of the control cohort Lifelines can be found on Github (https://github.com/molgenis/GAP). Genotype calling was performed with optiCall. SNP positions of one of the case cohorts were remapped from build 36 to build 37 (GRCh37, hg19) using liftOver (http://hgdownload.cse.ucsc.edu/admin/exe/linux.x86_64/). QC of genotype data was performed with PLINK (v1.9) using standard settings. Genoptye imputation was performed with the Sanger Imputation Server (https://www.sanger.ac.uk/tool/sanger-imputation-service/) using data from 1000Genomes phase 1 and the Haplotype Reference Consortium as reference panels. File formats were adapted using BCFtools (v1.16). Meta-analysis was performed with METAL using default settings. Clumping was performed with PLINK (v 1.9). A Manhattan plot was created with the GWASinspector package (version 1.5.1). Gene-based analysis through Functional Mapping and Annotation of Genome-Wide Association Studies (https://fuma.ctglab.nl/, v1.3.6). Regional association plots of each SNP were created with Locuszoom (v0.13.2). FINEMAP (v1.4.2) was used to identify the most likely causal variants of the meta-GWAS. Polygenic risk scores were calculated with SBayesRC (v0.2.0) and PLINK (v1.9) using default settings. Correlation (linkage disequilibrium) between SNPs was calculated with PLINK (v1.9). SNPs were annotated with ANNOVAR (version October 2019). Cell population relevant genes were studied using SNPsea and a heatmap of scRNAseq data was produced with the R-package heatmaply (version 1.3.0). Genetic correlations were calculated with LD score regression (LDSC) software using default settings. Colocalization analysis was performed with the coloc and the Sum of Single Effects (SuSie) R-packages using defaut settings and data from the (publically available) 1000 Genomes Phase 3 dataset.

For manuscripts utilizing custom algorithms or software that are central to the research but not yet described in published literature, software must be made available to editors and reviewers. We strongly encourage code deposition in a community repository (e.g. GitHub). See the Nature Portfolio guidelines for submitting code & software for further information.

Data

Policy information about availability of data

All manuscripts must include a data availability statement. This statement should provide the following information, where applicable:

- Accession codes, unique identifiers, or web links for publicly available datasets
- A description of any restrictions on data availability
- For clinical datasets or third party data, please ensure that the statement adheres to our policy

Data from FinnGen (v6), 1000Genomes (phase 1 and 3), and the Haplotype Reference Consortium were downloaded from each of their respective websites. SNPs of interest were extracted with VCFtools. GWAS Catalog database (version 21 April 2021) was used for in silico pleiotropy analysis. Single cell RNA sequencing data were aqcuired via correspondence. Blood cis-eQTL data were downloaded from the eQTLGen consortium. Fibroblast cis-eQTL data were downloaded from the Genotype-Tissue Expression (GTEx) version 8. Co-regulation analysis was performed using DEPICT and its accompanying expression dataset of 77,840 samples. The Phenoscanner database (version 2) was queried to look up quantitative trait loci (QTL) associations. GeneMANIA was used to construct composite networks of the prioritized genes based on the database accompanied by the software (build 12-02-2019). The STRING database v11.0 to find the protein-protein interactions. The Genotype-Tissue Expression (GTEx) database (v8) was used to study gene expression of prioritized genes. To assess enrichment of tissue-specific genes, the Human Protein Atlas (PMID 25613900) and mouse gene expression as well as RNAseq data from the GTEx database were used.

Research involving human participants, their data, or biological material

Policy information about studies with human participants or human data. See also policy information about sex, gender (identity/presentation), and sexual orientation and race, ethnicity and racism.

Reporting on sex and gender	Only the term sex was used in this study. For the genotype data, recorded (self reported) sex of individuals in the database and sex based on X chromosomes homo-/heterozygosity and updated sex according to genotype data was checked to detect sample switches.
Reporting on race, ethnicity, or other socially relevant groupings	In the article, the authors studied subjects of European ancestry. The ancestry was determined by the study participants. During genotype QC, non-European samples based on the first two principal components were removed.
Population characteristics	We meta-analysed existing data of six cohorts which have already been reported on before. We used data from six cohorts with Dupuytren's disease cases and healthy controls from the Netherlands, United Kingdom (UK), and Germany. The cases were individuals who had been diagnosed with DD by a plastic or hand surgeon and/or who had undergone surgical treatment for Dupuytren's disease. Controls were population-based subjects from the Lifelines cohort study (the Netherlands), from the UK Household Longitudinal Study (UK), the UK Biobank initiative (UK), and from the PopGen and KORA studies (Germany) with no known diagnosis of DD.
Recruitment	For this study, data of previous studies were used. The previous studies have reported on their methods of recruitment.
Ethics oversight	The studies used in this meta-analysis were approved by the Research Ethics Committee or equivalent at all institutions where the data were collected: 1) The Genetic Origin of Dupuytren Disease (GODDAF) Study (the Netherlands) was approved by the Ethics Committee of the University Medical Center Groningen, document number 2007/067; 2) The Lifelines study (the Netherlands) was approved by the Ethics Committee of the University Medical Center Groningen, document number 2007/152. This study ('The role of genetic variants in Dupuytren disease') has Lifelines study ID OV18_0461; 3) The British Society for Surgery of the Hand Genetics of Dupuytren's Disease (BSSH-GODD) study (United Kingdom) was approved by the Oxfordshire Research Ethics Committee, document number B/09/H0605/65; 4) The UK Biobank (United Kingdom) was approved by the North West Multi-Centre Research Ethics Committee, document number 11/NW/0382. This study ('The Genetics and Epidemiology of Common Hand Conditions') has UK Biobank study ID 22572; 5) The German Dupuytren Study was approved by the Ethics Commission of the Faculty of Medicine of the University of Cologne, document number 14/292.

Note that full information on the approval of the study protocol must also be provided in the manuscript.

Field-specific reporting

Please select the one below that is the best fit for your research. If you are not sure, read the appropriate sections before making your selection.

☒ Life sciences ☐ Behavioural & social sciences ☐ Ecological, evolutionary & environmental sciences

For a reference copy of the document with all sections, see nature.com/documents/nr-reporting-summary-flat.pdf

Life sciences study design

All studies must disclose on these points even when the disclosure is negative.

Sample size	We used all data available to date, to maximize the statistical power to detect associated variants.
Data exclusions	We excluded no data. We meta-analysed dataset that were already cleaned and published on.
Replication	We performed replication once, using freely available data from the FinnGen cohort, succesfully replicating 34 out of 85 SNPs identified in the meta-GWAS.
Randomization	We used all cases and controls available and corrected for covariates in the meta-analysis.
Blinding	Blinding is not applicable in genome-wide association studies as they study the genetic differences between cases and controls, and therefore it must be known which participants are cases or controls.

Reporting for specific materials, systems and methods

We require information from authors about some types of materials, experimental systems and methods used in many studies. Here, indicate whether each material, system or method listed is relevant to your study. If you are not sure if a list item applies to your research, read the appropriate section before selecting a response.

Materials & experimental systems

n/a	Involved in the study	
☒	☐	Antibodies
☒	☐	Eukaryotic cell lines
☒	☐	Palaeontology and archaeology
☒	☐	Animals and other organisms
☐	☒	Clinical data
☒	☐	Dual use research of concern
☒	☐	Plants

Methods

n/a	Involved in the study	
☒	☐	ChIP-seq
☒	☐	Flow cytometry
☒	☐	MRI-based neuroimaging

Clinical data

Policy information about clinical studies

All manuscripts should comply with the ICMJE guidelines for publication of clinical research and a completed CONSORT checklist must be included with all submissions.

Clinical trial registration	The studies meta-analysed in this research were not registered as clinical trails as they are cohort studies.
Study protocol	In the origional manuscript of the studies used in this research.
Data collection	In the origional manuscript of the studies used in this research.
Outcomes	NA

Plants

Seed stocks

Report on the source of all seed stocks or other plant material used. If applicable, state the seed stock centre and catalogue number. If plant specimens were collected from the field, describe the collection location, date and sampling procedures.

Novel plant genotypes

Describe the methods by which all novel plant genotypes were produced. This includes those generated by transgenic approaches, gene editing, chemical/radiation-based mutagenesis and hybridization. For transgenic lines, describe the transformation method, the number of independent lines analyzed and the generation upon which experiments were performed. For gene-edited lines, describe the editor used, the endogenous sequence targeted for editing, the targeting guide RNA sequence (if applicable) and how the editor was applied.

Authentication

Describe any authentication procedures for each seed stock used or novel genotype generated. Describe any experiments used to assess the effect of a mutation and, where applicable, how potential secondary effects (e.g. second site T-DNA insertions, mosiacism, off-target gene editing) were examined.
